# The Role of Omega-3 Polyunsaturated Fatty Acids in Modulating Inflammation and Breast Cancer: A Systematic Review of In Vitro, Animal, and Human Studies

**DOI:** 10.1007/s11912-026-01820-4

**Published:** 2026-08-21

**Authors:** Rahbika Ashraf, Connor D.C. Buchanan, Jennifer M. Monk, Lindsay E. Robinson, David W. L. Ma

**Affiliations:** Department of Human Health Sciences, University of Guelph, 50 Stone Road E, Guelph, ON N1G 2W1 Canada

**Keywords:** n-3 PUFA, Breast cancer, Inflammation, Eicosapentaenoic acid, Docosahexaenoic acid, Fish oil

## Abstract

**Purpose of Review:**

Inflammation is a hallmark of breast cancer (BC), influencing the tumor microenvironment (TME), immune evasion and tumor growth. N-3 polyunsaturated fatty acids (PUFA), particularly eicosapentaenoic acid (EPA) and docosahexaenoic acid (DHA), have anti-inflammatory and immunomodulatory effects, potentially mediated through anti-inflammatory and immune pathways. Evidence is varied by study design, dosage, and formulations, making it difficult to identify consistent mechanisms and therapeutic potential. Therefore, this systematic review aims to provide a comprehensive assessment of the biological and mechanistic effects of n-3 PUFA on inflammation in BC.

**Methods:**

A systematic search was conducted on PubMed, Web of Science, and the GOED (Global Organization for EPA and DHA) Clinical Study Database to identify preclinical and clinical studies investigating the effects of n-3 PUFA on inflammation and immune function in BC. Eligible studies were selected using predefined inclusion criteria (PROSPERO CRD42024499082) and assessed for risk of bias using Cochrane Handbook guidance. Primary outcomes included changes in cytokines, tumor-infiltrating immune cells, and inflammatory signaling pathways.

**Results:**

Of the 860 articles screened, 67 met the inclusion criteria. Across study types, n-3 PUFA demonstrated significant anti-inflammatory and anti-tumoral effects. Clinical studies showed IL-6 levels decreased by 50–70% with n-3 PUFA treatment. Preclinical studies reported significant reductions in proinflammatory cytokines (IL-6, TNF-α, CCL2/MCP-1), alongside increases in non-inflammatory cytokines (IL-10, IL-2) and a 57–90% reduction in lipid mediators (PGE_2_) and associated enzymes (COX-2) with n-3 PUFA treatment. In vitro findings showed large decreases (-40-80%) in NF-κB and matrix metalloproteinase (MMP-2, -9) activity with n-3 PUFA treatment. Variability in n-3 PUFA dose, form, and outcomes was noted across studies.

**Conclusions:**

n-3 PUFA exhibit anti-inflammatory and immunomodulatory effects in BC, highlighting their potential as adjuvant therapies to modulate the TME. Standardized research methodologies and clinical trials are needed to clarify dose-response relationships and therapeutic applications.

**Supplementary Information:**

The online version contains supplementary material available at 10.1007/s11912-026-01820-4.

## Introduction

Breast cancer remains the leading cause of cancer-related deaths among women worldwide with approximately 2.3 million diagnoses and an estimated 670,000 deaths reported in 2022 alone [1]. This disease also imposes a substantial economic burden with global costs projected to reach $2 trillion (Int$) between 2020 and 2050 [2]. The complexity of breast cancer arises from interactions between genetic, environmental, and lifestyle factors, with chronic inflammation playing a central role in its initiation and progression [3]. Within the tumor microenvironment (TME), inflammation fosters a supportive niche for tumor cells, enabling immune evasion, promoting angiogenesis and facilitating metastasis [4]. These processes are mediated through a network of cytokines, chemokines, and lipid mediators, making inflammation a promising target for therapeutic intervention.

N-3 polyunsaturated fatty acids (PUFA), predominantly found in fatty fish, marine oils and plant sources such as walnuts, chia seeds and flaxseed, have garnered attention for their anti-inflammatory and immunomodulatory properties, as well as their protective effects against various cancers including breast cancer [5–9]. Three major types of n-3 PUFA, eicosapentaenoic acid (EPA), docosahexaenoic acid (DHA) (from marine dietary sources), and the essential fatty acid precursor alpha-linolenic acid (ALA) (found in plant-based dietary sources), contribute to inflammation resolution, with EPA and DHA being metabolized into specialized pro-resolving mediators (SPMs) [10, 11]. Preclinical studies have demonstrated that n-3 PUFA and their mediators can modulate key signaling pathways, including nuclear factor-kappa B (NF-κB) and cyclooxygenase-2 (COX-2), thereby suppressing pro-inflammatory responses [9, 12–14]. Furthermore, the immunomodulatory properties of n-3 PUFA have been linked to enhanced antitumor immunity by modulating T-cell activation and promoting macrophage polarization toward an anti-inflammatory phenotype within the TME [14, 15]. Existing literature has highlighted reductions in inflammatory biomarkers, such as interleukin-6 (IL-6), tumor necrosis factor-alpha (TNF-α), and C-reactive protein (CRP), with n-3 PUFA supplementation [16–19]. However, inconsistencies persist, with some studies reporting null or contradictory effects, underscoring the need for systematic synthesis of evidence [20].

Despite these promising preclinical findings, challenges remain in translating the anti-inflammatory and anticancer potential of n-3 PUFA into clinical practice. Differences in study methodologies, including variations in PUFA sources, dosages, and durations of intervention, contribute to inconsistent outcomes [21]. In addition, the heterogeneity of mammary tumors represents a key source of biological variability that may influence responses to n-3 PUFA. Breast cancer comprises different molecular subtypes including hormone receptor-positive (e.g., luminal A and luminal B), human epidermal growth factor receptor (HER-2)-enriched and triple-negative, that may vary in metabolic wiring, transcriptomic profiles, immune infiltration and microenvironmental contexts [22]. Moreover, intra-tumoral heterogeneity (variability among cell clones within the same tumor) and non-genetic phenotypic plasticity (e.g., epithelial-mesenchymal transition, epigenetic reprogramming and metabolic adaptation) further diversify tumor cell states [23–25]. Differences in n-3 PUFA type and function also further complicate findings. For example, certain preclinical reports hint that the anti-cancer and anti-inflammatory effects of DHA may preferentially impact more aggressive subtypes (e.g., triple-negative breast cancer, TNBC), possibly due to high basal inflammation or lipid metabolism demands in those tumors [26–28]. Whereas, EPA may decrease cell viability to a greater extent in luminal B breast cancer cells compared to DHA [29, 30]. These sources of heterogeneity may differentially shape how a tumor and its microenvironment respond to n-3 PUFA interventions in both preclinical models and clinical settings. These diverse biological contexts underscore the need to interpret n-3 PUFA responses within the framework of tumor subtype and heterogeneity in breast cancer.

While previous meta-analyses and scoping reviews have investigated the effects of n-3 PUFA on inflammation in breast cancer, a comprehensive analysis encompassing preclinical and clinical findings remains limited [18, 31, 32]. Existing research has mainly focused on the role of n-3 PUFA in isolated contexts, including animal models or human trials, with limited systematic integration of findings across these research domains in breast cancer. This systematic review aims to address these gaps by evaluating the effects of n-3 PUFA on inflammatory markers in breast cancer across in vitro, animal, and human studies. By synthesizing evidence from different models and tumor subtypes, this review seeks to elucidate the therapeutic potential and gain a clearer understanding of underlying mechanisms through which n-3 PUFA modulate the inflammatory landscape of breast cancer. The insights gained may inform the design of future clinical trials, contributing to the development of evidence-based guidelines for incorporating n-3 PUFA into comprehensive breast cancer prevention, treatment, or management strategies. Ultimately, this work represents a critical step toward understanding the distinct and overlapping roles of ALA, EPA and DHA in breast cancer, with the potential to improve therapeutic outcomes, mitigate side effects associated with conventional treatments, and enhance patient quality of life.

## Methods

This systematic review adhered to the Preferred Reporting Items for Systematic Reviews and Meta-Analyses (PRISMA) guidelines [33] and was registered in the Prospective Registry of Systematic Reviews (PROSPERO) database under protocol number CRD42024499082.

### Search Strategy

A systematic search was conducted across PubMed, Web of Science, and the GOED Clinical Study Database [34] to identify relevant literature on n-3 PUFA and breast cancer. Search keywords included “n-3 PUFA”, “inflammation”, “immune system”, and “breast cancer”, combined with related Medical Subject Headings (MeSH) terms and Boolean operators to refine results. The search was restricted to original, full-text articles published in English up to November 2025. The detailed search strategy is provided in Supplementary Tables 1 and 2.

### Study Selection and Eligibility Criteria

All initial studies retrieved from searches were uploaded to Covidence systematic review software (Veritas Health Innovation, Melbourne, Australia). Following the removal of duplicate citations in Covidence, abstracts were independently screened by two reviewers to identify studies that met the inclusion criteria. Relevant full-text articles were subsequently assessed for eligibility based on predefined criteria. Peer-reviewed, published studies were included if they involved human, animal, or in vitro breast cancer models and provided interventions with n-3 PUFA, specifically EPA, DHA, or ALA. Eligible studies were required to report quantifiable outcomes related to inflammation or immune markers. Studies were excluded if they lacked clear intervention protocols, included male breast cancer cases, included mixed cancer types without appropriate subgroup analysis, or failed to report inflammatory outcomes. Additionally, observational human studies, reviews, editorials, case reports, preprints, abstracts and conference materials were also excluded from analyses.

### Data Extraction

Data extraction focused on key study characteristics, including design, population, sample size, intervention (source and dose of n-3 PUFA) and control details, and reported outcomes related to inflammation. Risk of bias was assessed using Cochrane’s RoB 2 tool for randomized clinical studies [35]. Extracted data were synthesized to identify patterns, gaps and significant results. Due to heterogeneity in study design, interventions, and outcome measures, a meta-analysis was not performed.

## Results

### Study Selection and Characteristics

**Fig. 1:**
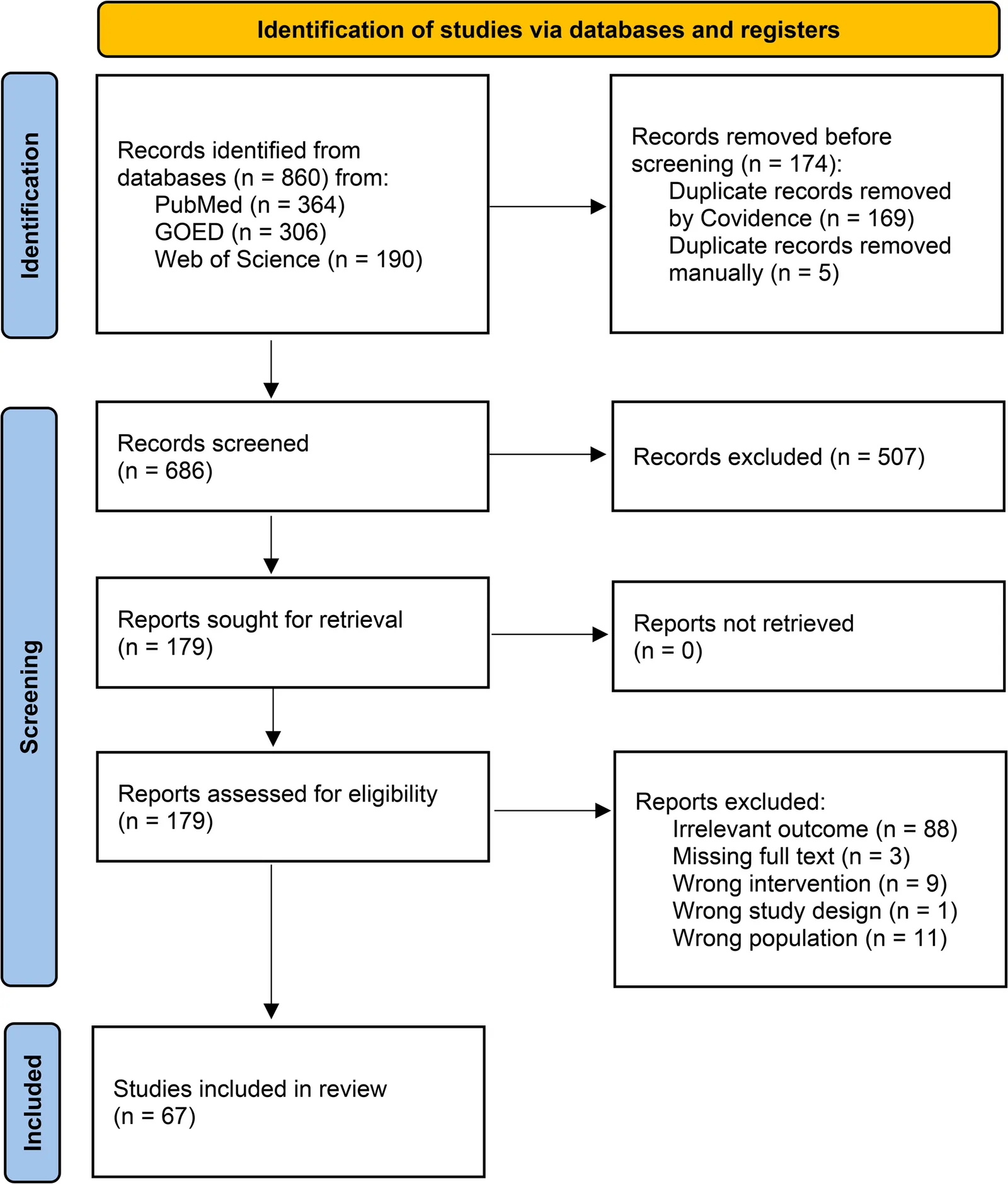
PRISMA flow diagram detailing the database searches and study selection process for the systematic review. (adapted from Page MJ et al., 2021) [33] by automation tools on Covidence. A total of 860 records were identified through electronic databases, including PubMed (*n* = 364), GOED (*n* = 306), and Web of Science (*n* = 190). After the removal of 174 duplicates, 686 records remained for title and abstract screening. Of these, 507 were excluded, and the remaining 179 full-text articles were assessed for eligibility. Ultimately, 112 full-text articles were excluded due to irrelevant outcomes (*n* = 88), missing full text (*n* = 3), wrong intervention (*n* = 9), wrong study design (*n* = 1), or wrong population (*n* = 11). A total of 67 studies were included in the final review. Screening and selection were performed using Covidence’s systematic review management tools

A total of 860 studies were identified through the systematic search. After removing 174 duplicates, 686 abstracts were screened, and 507 studies were excluded based on predefined criteria. Of the 179 full-text articles assessed for eligibility, 67 studies were included in the final analysis. This comprised 13 human studies, 29 animal studies, and 30 in vitro studies, with some studies contributing data across multiple categories. The PRISMA flow diagram summarizing the selection process is presented in Fig. 1.

### Overview of the Effects of n-3 PUFA in the Included Studies

#### Human Studies

The 13 included human studies demonstrated anti-inflammatory effects following n-3 PUFA supplementation in patients with breast cancer, however, variability in magnitude and outcomes persisted across studies **(**Table 1**)**. Dosing regimens in the clinical studies ranged from 1 to 4.4 g/day of n-3 PUFA supplementation, and intervention durations varied from 1 to 6 months. Commonly assessed markers across clinical studies included IL-6, CRP, and TNF-α.

**Table 1:** The characteristics of included studies investigating the effect of n-3 PUFA on biomarkers of inflammation in breast cancer: human studies

Author, Year, Country	Study participants and sample sizes (*n*)	Age (range, mean/median, years) (Intervention/Control)	Cancer subtype /stage	Duration	Concurrent treatment	Treatment vs. Control (Source/Dose/Duration)	Findings related to inflammation
Almassri et al., 2024 [43] Palestine	Women newly diagnosed with non-metastatic stage II-III BC, initiating chemotherapy (*n* = 88) Open-labelled randomized controlled trial	28–64 years	Stage I-III, HER-2-negative (HER-2–)	9 weeks	Adriamycin + Cytoxan (AC) chemotherapy (4 cycles; every 3 weeks)	600 mg/d n-3 PUFA with or without Vitamin D vs. control for 9 weeks; (i) ω-3 group: 2 × 300 mg capsules daily (180 mg EPA + 120 mg DHA per capsule; total 600 mg/day); (ii) Vit D group: 50,000 IU vitamin D₃ weekly; (iii) ω-3 + Vit D group: both interventions; (iv) Control: no supplement; (*n* = 22/group)	**Markers**: Serum protein hsCRP, TNF-α **Results**: TNF-α and hsCRP ↓ in ω-3 + Vit D group compared to baseline (*p* < 0.05) and hsCRP ↓ in ω-3 + Vit D vs. control after 9 weeks (*p* < 0.01); no significant change in TNF-α or hsCRP within ω-3 or Vit D groups alone. **Effect**: No effect of ω-3 alone on serum hsCRP or TNF-α.
Arsic et al., 2023 [36] Serbia	Non-metastatic, non-HER-2 + postmenopausal women with ER + and/or PR + BC, undergoing chemotherapy (*n* = 29; *n* = 14 Intervention; *n* = 16 Control) Double-blind, placebo-controlled, parallel-group study	45–70 years 56.14 ± 7.47 years/ 60.40 ± 9.19 years	ER + and/or PR+, HER-2–, stage IIa-IIIa	12 weeks	Adjuvant anthracycline	FO + evening primrose oil (FO, *n* = 14, (1000 mg EPA + DHA (400 mg EPA 600 mg DHA) + 351 mg GLA) or mineral oil placebo (*n* = 16) for 12 weeks	**Markers**: Plasma IL-10, IL-6, IL-8, TNF-α **Results**: IL-6 ↓ in both FO (30.67 to 9.42 ng/ml, *p* < 0.001) and placebo (33.79 to 12.76 ng/ml, *p* < 0.001); IL-6 was ↓ in FO group vs. placebo at 12 weeks (*p* < 0.01); no difference in TNF-α, IL-8 or IL-10. **Effect**: FO ↓ inflammation via lowering IL-6.
Darwito et al., 2019 [46] Indonesia	Patients with locally advanced (stage IIIB) IDC receiving standard neoadjuvant chemotherapy (*n* = 48; Intervention *n* = 24; Control *n* = 24) Double-blind randomized control trial	25–60 years 46.5 ± 8.07 years/ 48.5 ± 8.77 years	IDC, stage IIIb	51 days + 48 weeks follow-up	Neoadjuvant CAF	1 g/day FO (*n* = 24) or placebo (*n* = 24) for 51 days with median follow-up period of 48 weeks	**Markers**: Tumor Ki-67 and VEGF protein expression **Results**: FO ↓ Ki-67 (42.4 ± 4.8 vs. 39.2 ± 5.3, *p* = 0.032) and VEGF (32.7 ± 5.2 vs. 29.5 ± 5.4, *p* = 0.041) compared to placebo. **Effect**: FO ↓ angiogenesis and cell proliferation.
Gucalp et al., 2018 [20] USA	Women with overweight/obesity (BMI ≥ 25 kg/m²) with history of stage I-III BC, DCIS, LCIS, Paget’s disease, or proliferative benign breast disease (*n* = 62; Intervention *n* = 32; Control *n* = 32) Randomized, placebo-con-trolled, double-blind phase II study	Median age 59 (IQR: 52–62) years	DCIS/LCIS/stage I-III	12 weeks	None	2 g/day of DHA (algal-derived, *n* = 32) or placebo (*n* = 32) for 12 weeks	**Markers**: Breast tissue gene expression of TNF-α, COX-2, IL-1β, aromatase, and WAT inflammation (CLS-B) **Results**: DHA ↔ in inflammatory biomarkers compared to baseline or placebo (*p* > 0.05). **Effect**: No effect of DHA on breast tissue inflammation.
Hershman et al., 2015 [44] USA	Postmenopausal women with early-stage HR + BC experiencing AI-induced joint pain (*n* = 249; Intervention *n* = 122; Control *n* = 127). Randomized multicenter placebo-controlled trial	Median age ~ 59 years	ER + and/or PR+, stage I-III	24 weeks	Adjuvant hormonal therapy with AI (Anastrozole, Exemestane, Letrozole)	3.36 g/d n-3 PUFA (~ 2.24 g/d EPA + 1.12 g/d DHA, *n* = 122) or soybean/corn oil placebo (*n* = 127) for 24 weeks *n* = 85 and *n* = 90 for CRP analyses for n-3 and placebo group, respectively.	**Marker**: Serum CRP **Results**: n-3 PUFA ↔ CRP levels compared to baseline or placebo. **Effect**: No anti-inflammatory effect for n-3 PUFA.
Hutchins-Wiese et al., 2014 [40] USA	Postmenopausal women receiving AI for treatment of ER + BC for ≥ 6 months (*n* = 38; Intervention *n* = 20; Control *n* = 18) Randomized, double-blind, placebo-controlled pilot study	48–84 years Mean age 62 years 60.9 ± 10.5 years/ 63.6 ± 8.6 years	ER+	3 months	Adjuvant hormonal therapy with AI	7 capsules/d containing 4 g/d of EPA + DHA FO (2520 mg EPA and 1680 mg DHA) or safflower oil placebo (9% linoleic acid, 83% oleic acid) for 3 months	**Markers**: Serum hsCRP, IL-6, IL-1β, TNF-α **Results**: FO ↑ hsCRP (2.86 3.29 to 4.79 ± 6.59, *p* = 0.027) compared to baseline. No significant changes in other markers. Secondary analysis with outliers removed continued to show differences in hsCRP change (FO: 7.78 ± 8.38 vs. placebo: 2.38 ± 3.25, *p* = 0.022). **Effect**: FO did not show consistent effects on inflammatory markers.
Lustberg et al., 2018 [37] USA	Postmenopausal women with ER + or PR+ stage I-III breast cancer, initiating AI therapy (*n* = 44; Intervention *n* = 22; Control *n* = 22) Randomized, double-blind, placebo-controlled study	43–76 years Mean age 59.5 ± 8.1 years	ER + or PR+, stage I-III	24 weeks	Adjuvant hormonal therapy with AI (Anastrozole, Exemestane, Letrozole)	4.3 g/d n-3 PUFA (2580 mg/d EPA + 1380 mg/d DHA TGs) or placebo (mixture of fats and oils) for 24 weeks	**Markers**: Serum IL-6, TNFR-2, IL-17 **Results**: n-3 PUFA ↓ IL-6 (change, week 0–24: **-**0.25 ± 0.10, *p* = 0.02) but differences between the groups were not significant. Greater ↓ in IL-6 were observed within the n-3 group among patients with prior chemotherapy. ↔ in TNFR-2 or IL-17. **Effect**: n-3 PUFA ↓ IL-6 from baseline only; no effect on TNFR-2 or IL-17.
Mantha et al., 2022 [38] France	Women aged > 18 years with advanced HER-2–, HR + BC, starting first-line chemotherapy (*n* = 46; Intervention *n* = 21; Control *n* = 25) Randomized, double-blind, controlled, phase III clinical trial	60.6 ± 2.1 years/ 57.0 ± 2.1 years (mean SEM)	Metastatic HER-2–, HR+	3 months	Chemotherapy	3 cans/d FO (2.64 g/d EPA + 1.56 g/d DHA + 1.5 g/d ALA) or copra oil placebo for 3 months	**Markers**: Plasma IL-6 and TNF-α **Results**: IL-6 ↓ after 3 months (*p* < 0.05), 20:4n6 ↓ after 10 days, and ↔ in TNF-α in FO compared to baseline. **Effect**: FO ↓ inflammation via IL-6 and 20:4n6.
Martínez et al., 2019 [42] Spain	Post-menopausal women with early-stage (0-IIIA) ER + and/or PR + BC, currently receiving adjuvant hormonal therapy (*n* = 45) Prospective, multicenter, open-label, single arm, clinical trial	40–81 years Median age 57 years	ER + and/or PR+, stage 0-IIIa	1 month + 1 month follow-up	Adjuvant hormonal therapy (67% received AI and 33% tamoxifen)	3 capsules/d FO, (providing 1380 mg/d EPA + DHA, 37.5 mg/d hydroxytyrosol, and 150 mg/d curcumin) for 1 month	**Markers**: Peripheral blood CRP, SAA, IFN-γ, TNF-α, IL-10, IL-15, TGFβ, IGF-1 levels **Results**: CRP ↓ 18.5% after 1 month of FO (8.2 ± 6.4 to 5.3 ± 3.2 mg/L; *p* = 0.014), with a sustained ↓ 1 month post-intervention (*p* = 0.064). ↔ in IFN-γ (*p* = 0.056) or other cytokines after 1 month. **Effect**: FO ↓ inflammation via CRP only.
Molfino et al., 2025 [47] Italy	Women newly diagnosed with primary breast cancer (*n* = 26) and women with benign breast disease as controls (*n* = 8); all treatment-naïve Single-center, interventional, controlled study	47.4 ± 8.7 years (BC)/ 47.3 ± 5.4 years (controls)	Mixed subtypes: Luminal A (42.3%), Luminal B (3.8%), HER-2+ (30.8%), TNBC (23.1%); 76.9% high Ki67 ≥ 20%	10 days	None (treatment-naïve prior to any therapy)	Oral DHA supplementation (2 g/day as algal oil syrup; Schizochytrium sp.) for 10 days or control (benign breast disease group, no supplement)	**Marker**: Plasma (secreted protein) RvD1 and RvD2 **Results**: ↔ in RvD1 or RvD2 after DHA in pooled BC group or controls; BRCA1/2-mutated patients: DHA ↑ RvD1 (+ 185.8%, *p* = 0.037) and ↑ RvD2 (+ 101.2%, *p* = 0.028); Familial BC group: DHA ↓ RvD1 (–73.6%, *p* = 0.015) compared to baseline; Low Ki67 group (< 20%) showed greater ΔRvD2 with DHA compared to high Ki67 expression group (*p* = 0.046). **Effect**: DHA modulated plasma pro-resolving lipid mediators, enhancing inflammation-resolving capacity in BRCA1/2-mutated patients while familial cases showed a blunted response.
Munhoz et al., 2025a [41] Canada	Women with locally advanced breast cancer undergoing neoadjuvant chemotherapy (*n* = 49; DHA *n* = 23; placebo *n* = 26) Randomized, double-blind, placebo-controlled trial	33–65 years Mean age ~ 50 years	HER-2 + and HER-2–	18 weeks (baseline–week 15– surgery)	Neoadjuvant chemotherapy (HER-2–: fluorouracil + epirubicin + cyclophosphamide; HER2+: docetaxel + carboplatin + trastuzumab)	11 capsules/d DHA-enriched algal oil (4.4 g/day, TGs; Life’s DHA S40-O400; 43.4% DHA) from first chemotherapy cycle to surgery (~ 18 weeks) or corn/soy oil placebo capsules (isocaloric, matched in appearance/dose)	**Markers**: Plasma cytokines and chemokines (secreted protein; IFN-γ, TNF-α, IL-17 A, IL-17B, IL-21, IL-27, IL-2, IL-4, IL-6, IL-8, IL-10, IL-12, IL-1β, TGF-β1, MIP-3α) CRP, MMP-2, MMP-9; ex vivo secreted oxylipins (unesterified forms in PBMC supernatant following LPS stimulation); tumor tissue CD4^+^/CD8^+^ (protein) infiltration **Results**: DHA ↑ IFN-γ (0.64 to 1.55 pg/mL, *p-*interaction = 0.002) and TNF-α (0.88 to 1.17 pg/mL, *p-*interaction = 0.033) at week 15 vs. placebo; ↑ IL-17 A (0.58 to 1.05 pg/mL, *p* = 0.02); ↔ in Th1/Th2 ratio in DHA vs. ↓ in placebo (*p* < 0.05); ↓ TGF-β1 (8.58 to 4.99 ng/mL; *p* = 0.005); ↔ in CRP, MMPs, CD4^+^, CD8^+^, CD4/CD8 or other cytokines vs. placebo; ↑ total n-6 (583 to 914 ng/mL, *p* = 0.006) and total AA-derived (343 to 537 ng/mL, *p* = 0.043) vs. placebo; ↑ 15-HETE, 13-HODE and n-3-derived 13-HDoHE vs. placebo (*p* < 0.05). **Effect**: DHA enhanced circulating Th1 cytokines (IFN-γ, TNF-α), preserved Th1/Th2 balance, and increased oxylipin mediator production, suggesting immunostimulatory modulation of systemic inflammation.
Munhoz et al., 2025b [45] Canada	Women with locally advanced breast cancer undergoing neoadjuvant chemotherapy (*n* = 49; DHA *n* = 23; placebo *n* = 26) Randomized, double-blind, placebo-controlled trial	33–65 years Mean age 50.6 ± 8.2 years	HER-2 + and HER-2– (HER-2+, TNBC, Luminal A and Luminal B), stage II–III	18 weeks (baseline–week 15– surgery)	Neoadjuvant chemotherapy (HER-2–: fluorouracil + epirubicin + cyclophosphamide; HER2+: docetaxel + carboplatin + trastuzumab)	11 capsules/d DHA-enriched algal oil (4.4 g/day, TGs; Life’s DHA S40-O400; 43.4% DHA) from first chemotherapy cycle to surgery (~ 18 weeks) or corn/soy oil placebo capsules (isocaloric, matched in appearance/dose)	**Markers**: Cytokines (secreted proteins in isolated PBMC supernatant following PHA or LPS stimulation; IFN-γ, TNF-α, IL-2, IL-4, IL-6, IL-8, IL-10, IL-17, IL-21, IL-1β and TGF-β1); stimulated T cells in PBMC (CD3^+^, CD4^+^, CD8^+^, CD4/CD8, Tregs, Th1) **Results**: In PHA-stimulated PBMCs DHA attenuated declines in IL-4, IL-10, and IFN-γ (*p*-interaction < 0.05) vs. placebo; ↑ IL-17 A (*p* = 0.008) vs. baseline; In LPS-stimulated PBMCs DHA ↑ TNF-α and IFN-γ secretion (*p*-interaction < 0.05) vs. placebo; ↔ in proportion of total T cells (CD3^+^) in DHA vs. ↓ in placebo (*p*-interaction = 0.029); ↔ in other cytokines or T cell populations compared to placebo. **Effects**: DHA enhanced PBMC cytokine secretion capacity (Th1/Th17 cytokines: IFN-γ, TNF-α, IL-17) and maintained immune balance.
Paixão et al., 2017 [39] Brazil	Newly diagnosed treatment-naïve BC patients with BI-RADS 4 C or higher mammographic classifications, and surgery as the primary treatment option (*n* = 37; Intervention *n* = 18; Control *n* = 19) Randomized, controlled, double-blind study	18–70 years 48.6 years/ 53.4 years	Mixed subtypes: predominantly ER+	30 d	None	2 g/d FO (940 mg/d EPA + 780 mg/d DHA EEs) or mineral oil placebo for 30 d	**Markers**: Serum hsCRP, peripheral blood CD4 + and CD8+, plasma IL-6, IL-1β, TNF-α and PGE_2_ **Results**: FO maintained hsCRP levels, while placebo ↑ hsCRP (*p* = 0.024; between-group *p* = 0.059). CD4 + T-cells (%) remained stable with FO but ↓ with placebo (*p* = 0.042). No changes in other cytokines or PGE_2_. **Effect**: FO modulated inflammation by maintaining hsCRP and CD4 + T-cell levels.

↑, Higher than control/baseline; ↔, No difference from control/baseline; ↓, Lower than control/baseline. Unless otherwise specified

A double-blind randomized controlled trial (RCT) conducted in postmenopausal women undergoing chemotherapy for non-metastatic estrogen receptor (ER)-positive/progesterone receptor (PR)-positive (ER+/PR+) breast cancer reported a 70% reduction in IL-6 levels (30.67 to 9.42 ng/ml) after 12 weeks of supplementation with 1 g/day EPA and DHA combined with evening primrose oil (*p* < 0.001) [36]. Similarly, reductions in IL-6 levels were observed in two other studies following 3 to 6 months of n-3 PUFA supplementation with one study showing more pronounced reductions in patients who had undergone chemotherapy [37, 38]. In contrast, three studies found no significant effects on serum or plasma IL-6 levels. These included one in newly diagnosed patients not receiving chemotherapy who were given 2 g/day fish oil (FO) in the form of EPA and DHA ethyl esters (EEs) for 30 days, another in postmenopausal women with ER+ breast cancer receiving aromatase inhibitor (AI) therapy and 4 g/d of FO (2.25 g EPA and 1.68 g DHA) supplementation for 3 months, and a third in women undergoing neoadjuvant chemotherapy supplemented with 4.4 g/day DGA for 18 weeks [39–41].

The effects of n-3 PUFA on circulating CRP or high sensitivity (hs) CRP also varied. One study reported a significant reduction (-18.5%) in CRP levels after 1 month of supplementation with fish oil (1380 mg/day EPA + 37.5 mg/day DHA) [42], and another found significant decreased in hsCRP (*p* < 0.01) following co-supplementation with 600 mg/day n-3 PUFA (360 mg EPA + 240 mg DHA) and vitamin D [43], while others found no significant effects [39, 41, 44]. Other inflammatory markers, such as TNF-α, IFN-γ, IL-10 and IL-1β were also evaluated. While there was a trend toward reductions in TNF-α [36, 38] and IFN-γ [42], other studies reported inconsistent findings [20, 39], where these inflammatory cytokines were not significantly altered. Changes in IL-10 and IL-1β levels were generally less pronounced or non-significant [20, 36, 39–42]. However, one study reported that 4.4 g/day DHA supplementation during neoadjuvant chemotherapy attenuated the decline in secreted IL-10 levels in phytohemagglutinin (PHA)-stimulated peripheral blood mononuclear cells (PBMCs) [45].

**Fig. 2:**
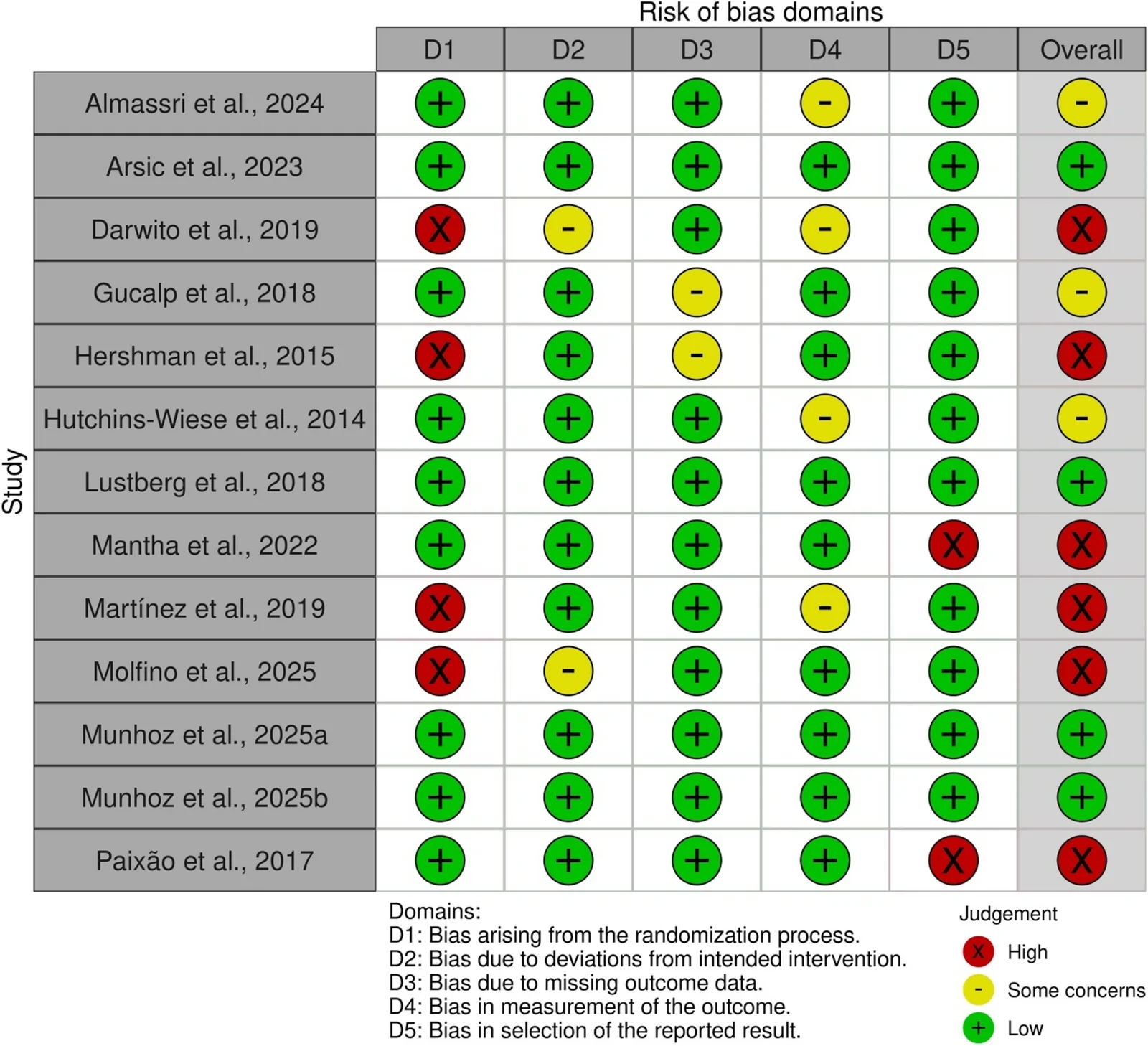
Risk of bias assessment of included RCTs using guidance from the Cochrane Risk of Bias Tool for Randomized Trials (RoB 2) [35]. Judgments were categorized as “Low risk”, “Some concerns” or “High risk” across the five domains. The overall risk of bias for each study was determined according to the RoB 2 algorithm, where the most severe domain-level judgment governs the overall rating. Specifically, a study is rated “High risk” if at least one domain is high risk, or if multiple domains raise some concerns; “Some concerns” if one or more domains raise concerns but none are high risk; and “Low risk” only if all domains are rated low risk

**Fig. 3:**
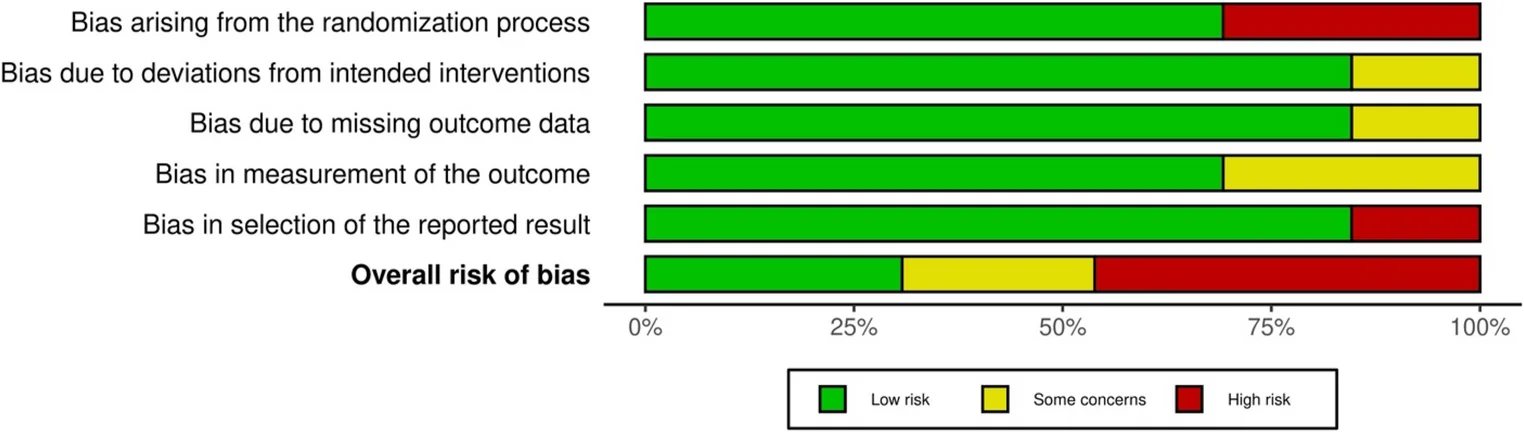
Risk of bias assessment of each domain presented as percentages across all included humans studies [35]

Risk of bias was assessed using the Cochrane RoB 2 tool [35] across five domains for each included study **(**Fig. 2**)**. Of the 13 trials, four [36, 37, 41, 45] were rated as low risk of bias across all domains, indicating high methodological quality. Three studies [20, 40, 43] had some concerns, and six were judged to have high risk of bias [38, 39, 42, 44, 46, 47]. Domains most frequently rated as high or unclear included bias arising from the randomization process [42, 44, 46, 47], and bias in the selection of the reported result [38, 39] **(**Fig. 3**)**. Common contributors to elevated risk of bias included lack of allocation concealment, absence of pre-registered protocols or statistical analysis plans, unblinded outcome assessment, and in two cases, the use of non-randomized or single-arm study designs [42, 47], which was automatically rated as high risk.

#### Animal Studies

The 29 animal studies collectively reported reductions in pro-inflammatory or increases in anti-inflammatory markers following n-3 PUFA supplementation **(**Table 2**)**. n-3 PUFA supplementation reduced tumor mRNA expression and plasma TNF-α and IL-6 levels across studies [5, 48–50], while others found no significant changes in these markers in tumors or plasma [51, 52]. Reductions in CCL2 (also referred to as monocyte chemoattractant protein, (MCP)-1) were also observed [9], although some studies reported no changes [12, 53]. Prostaglandin E_2_ (PGE_2_) is a bioactive lipid-derived mediator generated through COX-2 metabolism of n-6 PUFA arachidonic acid (AA) and is involved in promoting inflammation, angiogenesis and tumor progression [54, 55]. Plasma and tumor PGE_2_ levels significantly decreased with n-3 PUFA supplementation in multiple studies [12, 49, 56, 57], with one study reporting a -90% reduction [58] in metastatic mammary MX-1 tumor cells, a model of triple-negative breast cancer (TNBC). However, another study reported no change in PGE_2_ levels [59].

**Table 2:** The characteristics of included studies investigating the effect of n-3 PUFA on biomarkers of inflammation in breast cancer: animal studies

Author, Year, Country	Animal model	Type of model/tumor	Age at start (weeks)	Concurrent treatment	Treatment vs. Control (Source/Dose/Duration)	Findings related to inflammation
Chen et al., 2021 [56] Canada	Female Sprague-Dawley rats	Mammary tumor induction by drug (MNU)	3 weeks (21 d)	None	DHA (1.5 mg/kg BW, twice weekly by oral gavage) vs. control (20% corn oil (CO), modified AIN-76 A) for 7 weeks (*n* = 25/group total; subset *n* = 6/group for PGE_2_ analyses)	**Marker**: MG and tumor tissue PGE_2_ secretion **Result**: DHA ↓ PGE_2_ in non-involved MG (2.8 vs. 15 nM) and tumors (18 vs. 41 nM) compared to CO (*p* < 0.05). DHA ↓ mammary tumor multiplicity (DHA: 1.69 ± 0.26 vs. control: 2.55 ± 0.32 tumors/rat, *p* < 0.05). **Effect**: DHA ↓ pro-inflammatory mediator, PGE_2_.
Chung et al., 2015 [85] USA	OVX C57BL/6J mice (WT); Py230 mammary tumor cells derived from PyVmT/C57B mouse	Induction by Py230 tumor cells (orthotopic) injection into mammary fat pads; obesity-induced by HFD	7–8 weeks	None	Isocaloric HFD-FO (30% w/w; 24% DHA, 6% EPA) vs. standard HFD (60% kcal fat; D12492) for 22 weeks (*n* = 6–8/group for subset analyses)	**Markers**: F4/80, CD11c, CCL2/MCP-1, TNF-α, IL-6, IL-1β, iNOS, ARG-1 gene expression in mammary fad pad, perigonadal fat and mammary tumors **Result**: FO ↓ macrophage markers (F4/80, CD11c) and cytokines (CCL2/MCP-1, TNF-α) and ↑ IL-6 (all *p* < 0.05) in perigonadal fat and mammary fat pad in tumor naïve mice. FO ↑ ARG-1 in mammary fat pad only. FO generally showed non-significant ↑ in macrophage and inflammatory cytokines in mammary fat pad of tumor-bearing mice. Mammary tumor inflammation ↔ in FO vs. HFD diet. **Effect**: FO ↔ inflammatory mediators in tumor-bearing mice.
Colombo et al., 1997 [58] USA	Female BALB/c athymic nude (nu/nu) mice	Induction by human mammary carcinoma MX-1 tumor cells (subcutaneous implantation); TNBC	Not specified	HePC administered orally at doses of 200 µmol/kg every 4–5 days or 45 µmol/kg/d	MaxEPA FO (10% w/w) ± HePC for 30 d vs. corn oil (CO, 10% w/w in AIN-76 A diet); *n* = 10/group; *n* = 4/group for subset analyses	**Markers**: Tumor PGE_2_ secreted levels **Results**: FO ↓ PGE_2_ (–90%) alone or in combination with HePC (*p* < 0.001) **Effect**: FO ↓ pro-inflammatory mediator, PGE_2_.
Connolly et al., 1999 [57] USA	Female athymic nude mice (NCr-*nu/nu)*	Induction by cell (MDA-MB-231) injected into thoracic mammary fat pads; TNBC	3–4 weeks	None	DHA (2% or 4% DHA + 4% LA) from microalgae in TG form vs. LA (4% or 8%) for 12 weeks post-tumor cell injection (*n* = 20–50/group)	**Marker**: Tumor PGE_2_, 12-HETE, and 15-HETE production **Result**: DHA (2% or 4%) ↓ PGE_2_, 12-HETE, and 15-HETE compared to 4% LA (*p* < 0.001). **Effect**: DHA ↓ pro-inflammatory tumor eicosanoids.
Dyari et al., 2017 [86] Australia	Female Balb/c nu/nu mice with MDA-MB-231 cell xenografts	Induction by cell (MDA-MB-231) injected into left inguinal MG; TNBC	6-weeks	None	C20E (0.05 or 0.5 mg/kg in corn oil containing 6% DMSO; 50 µl) + AUDA (10 mg/kg, IP) 1x/day (5 d/wk) for 42 d vs. vehicle control (*n* = 4–8/group)	**Marker**: Tumor apoptosis (TUNEL), Ki67 protein expression, tumor growth **Result**: 0.05 and 0.5 mg/kg C20E+AUDA ↓ tumor Ki67 staining, ↑ TUNEL staining in xenograft tumors and ↓ relative tumor: BW and tumor vol: BW (*p* < 0.05). **Effect**: C20E ↓ tumor cell proliferation, ↑apoptosis and ↓ tumor growth.
El-Mesery et al., 2009 [87] Egypt	Female Swiss albino mice (20–30 g)	Induction by EAC cells	Not specified	Cisplatin (CP, 5 mg/kg, IP) + DHA	DHA (125 and 250 mg/kg), orally administered for 20 days vs. control or CP (*n* = 8–10/group)	**Markers**: Serum CRP, leukocytic count and MDA **Results**: DHA (250 mg/kg) ↓ CRP and ↓ leukocytic count to baseline levels (*p* < 0.05). DHA (125 and 250 mg/kg) ↓ MDA (*p* < 0.05). **Effect**: DHA (alone or in combination with CP) dose-dependently ↓ systemic inflammation, leukocytosis and oxidative stress, highlighting its potential as a chemo-enhancer.
Elisia et al., 2022 [49] Canada	Female C3(1)/Tag mice (hemizygous)	Transgenic, spontaneous mammary adenocarcinomas; TNBC	5 weeks; euthanized at 20–22 weeks	None	FO + low-CHO (15% Amylose/Soy/FO) for 22 weeks vs. Western diet (*n* = 13; *n* = 5–11 for cytokine/marker subset analyses)	**Markers**: Plasma and tumor cytokines (TNF-α, IL-6, IFN-γ, IL-1β, IL-12p70), PGE_2_ ,VEGF, Ki67 **Result**: FO ↓ plasma TNF-α, IL-6, PGE_2_ and tumor Ki67 (*p* < 0.05). ↔ in IFN-γ, IL-1β, IL-12p70, or VEGF. **Effect**: FO ↓ inflammatory cytokines and cell proliferation.
Ford et al., 2015 [12] USA	Female OVX C57BL/6 mice	Induction by Wnt-1 and M-Wnt tumor cells (orthotopic); TNBC (Basal-like and Claudin-low)	10 weeks	None	EPA + DHA EEs in 10% or 60% kcal fat diets (0.025% of diet, equivalent to 4 g/day Lovaza in humans) for 10–15 weeks vs. controls: 10% (standard diet) or 60% kcal fat (diet-induced obesity, DIO) (*n* = 92; *n* = 23/group)	**Marker**: Tumor COX-2, NF-κB P-p65, PGE_2,_ PGE_3_ and serum adipokines, TNF-α and CCL2/MCP-1 **Results**: EPA + DHA ↓ COX-2, NF-κB P-p65, PGE_2_, leptin/adiponectin (-40%), and ↑ PGE_3_ and cleaved caspase-3 (*p* < 0.05). ↔ in TNF-α and CCL2/MCP-1. **Effect**: EPA + DHA mitigated pro-inflammatory effects of the DIO diet.
Goupille et al., 2020 [53] France	Female Sprague-Dawley rats	Mammary tumor induction by drug (MNU 25 mg/kg)	6 weeks (48 d)	Docetaxel (6 mg/kg/week) after tumors reached 2 cm^3^; *n* = 14/group) for 6 weeks	5% FO (2.5% DHA, 1% EPA) + 8% peanut and 2% rapeseed oil vs. control (12% peanut + 3% rapeseed oil) (*n* = 28/group; *n* = 14/group for subset analyses) for 6 weeks	**Markers**: Tumor mRNA AREG, EREG, TNF-α, MMP-2,3,9, VEGF-β, CXCL1,2,9, plasma VEGF-α **Result**: FO ↑ tumor microvascularization and regression in response to docetaxel; ↓ proangiogenic genes (EREG and AREG) (*p* < 0.05). ↔ in VEGF-α/β, TNF-α, MMPs or CXCL. **Effect**: FO ↓ pro-inflammatory angiogenesis markers and enhanced tumor vascular remodeling.
Guo et al., 2021 [88] Taiwan	Female BALB/c mice	Induction by 4T1 tumor cells (injected into right hind thigh); TNBC	8 weeks	Avastin (bevacizumab)	Low FO (5.1 mg/g EPA + 3.7 mg/g DHA), medium FO (9.1 mg/g EPA + 6.9/g mg DHA) or high FO (10.7/g mg EPA + 8.3/g mg DHA) + Se (oral gavage 2x/d) + Avastin (5 mg/kg, IP, every 4 days) for 24 d vs. Avastin alone (5 mg/kg, IP, every 4 days) or saline (0.9% NaCl, control) (*n* = 8/group)	**Markers**: Tumor VEGF/phospho-VEGFR2, HIF-1α, HIF-2α, COX-2, MMP-9, FGFR, EGF, TGFβ, TGFβR, CXCL12, CXCR4,7 protein expression **Results**: FO ↓ VEGF/phospho-VEGFR2, HIF-1α and HIF-2α, COX-2, MMP-9, TGFβ, TGFβR2 and CXCL12/CXCR4,7 dose-dependently (*p* < 0.05). FO ↑ cleaved-caspase-3, -8. ↔ in FGFR, EGF, or TGFβR1. **Effect**: FO + Se with Avastin ↓ pro-inflammatory and angiogenesis-related markers.
Guo et al., 2021 [48] Taiwan	Female BALB/c mice	Induction by 4T1 tumor cells (injected into right hind thigh); TNBC	8 weeks	Taxol (5 mg/kg, IP), Adriamycin (2 mg/kg, IV), or Avastin (5 mg/kg, IP) every 4 d	Nutritional supplement (5.1 mg/g EPA + 3.7/g mg DHA in TG form) + Se supplement (oral gavage, 2x/d) ± anti-cancer drugs for 24 d vs. healthy/tumor-bearing controls (saline) or anti-cancer drugs alone (*n* = 8/group)	**Markers**: Plasma IL-1β, IL-6, IL-10, TNF-α, IFN-γ, IL-2, VEGF secreted levels; Tumor VEGF, CD31, MMP-9, HIF-1α, MDA, CD24 and CD29 protein expression **Results**: EPA/DHA + Se ↓ IL-1β, IL-6, IL-10, TNF-α, VEGF, CD31, MMP-9, HIF-1α, CD24, CD29; ↑ IFN-γ, IL-2, MDA, PD-1, cleaved-caspase-3 (*p* < 0.05). Combination treatment with anti-cancer drugs enhanced these effects. **Effect**: EPA/DHA + Se ↓ tumor progression, metastasis, angiogenesis, hypoxia, and cancer stem cell markers; modulated immune responses, and ↑ apoptosis.
Hamid et al., 1999 [89] USA	Female inbred F344 rats	Mammary tumor induction by drug MNU (37.5 mg/kg body weight, IV)	4 weeks (standard diet from 4–7 weeks (28–50 d); treatment from 51 d onward)	None	18% menhaden oil (FO) + 5% corn oil (CO) for 31 weeks vs. 5% CO, 18% CO + 5% FO, or 23% CO (*n* = 6/group)	**Markers**: Tumor COX-1 and − 2 protein expression **Results**: 18% FO ↓ COX-1 (–28%, *p* < 0.05) and − 2 (–36%, *p* < 0.01) compared to high n-6 diet (23% CO). **Effect**: MO ↓ MNU-induced mammary tumor eicosanoid levels.
Ion et al., 2021 [90] USA	Female hemizygous C(3)1 TAg transgenic mice (SV129 x C(3)1 TAg)	Transgenic	3 weeks	None	5% w/w FO + canola oil for 120–140 d of age vs. 10% w/w corn oil (CO) (*n* = 15–17/group); offspring maintained on maternal diets or switched	**Markers**: MG TNF, CCL2/MCP-1, CD5, CCL20, CSF-2, IL-2, IL-2R, IL-4Rα, Lef1, Lta gene expression, tumor incidence and multiplicity **Results**: Maternal FO ↑ CCL20, CD5, IL-2, Lef1, Lta, TNF, WNT1. **Effect**: Lifelong/maternal FO ↑ immune system activation; ↓ tumor incidence and multiplicity.
Khadge et al., 2018 [52] USA	Female BALB/c mice	Induction by 4T1 mammary tumor cells (orthotopic) in left 5th MG	6 − 8 weeks	None	FO (modified Lieber-DeCarli; corn, olive, safflower and fish oils) for 10 − 16 weeks vs. *n* − 6 PUFA (Lieber-DeCarli; corn, olive and safflower oils) control diet (*n* = 10/group for subset analyses)	**Markers**: Tumor immune cell infiltration (CD3+, F4/80+, NE+), IL-10, NF-κB, TNF-α, CCL2/MCP-1, CXCL1,CXCL5, GM-CSF, AREG) mRNA expression, tumor Ki67 + cells, tumor volume, metastatic spread. **Results**: FO ↓ tumor and contralateral MG F4/80+; ↓ tumor NE+, NLR, Ki67 + cells, tumor volume (− 50.2%) and metastatic spread; ↑ CD3 + T-cells and tumor IL-10 mRNA (*p* < 0.05). ↔ in NF-κB, TNF-α, CCL2, GM-CSF, G-CSF and AREG. **Effect**: FO modulated the tumor microenvironment by ↓ proinflammatory cell infiltration and ↑ anti-inflammatory immune responses associated with ↓ metastasis.
Liu et al., 2018 [9] Canada	Female FVB MMTV-neu(ndl)-YD5 mice	Transgenic; HER-2+	In utero; postnatally from 3 to 20 weeks	None	3% Menhaden oil (FO; EPA + DHA) and 3% or 10% flaxseed oil (FS; ALA) for 20 weeks vs. 10% safflower oil (SO); *n* = 12/group (tumor development); *n* = 6–10/group (subset analyses)	**Markers**: Tumor 5-LOX, COX-2, NF-κB, TNF-α, CCL2/MCP-1, IL-6 gene expression, Ki-67 tumor latency, volume, multiplicity **Results**: FO ↓ 5-LOX, COX-2, NF-κB, TNF-α, CCL2/MCP-1 expression (*p* < 0.05). 10% FS ↓ NF-κB and IL-6 (*p* < 0.05). n-3 PUFA (FO and FS) ↓ Ki-67, delayed tumor onset and ↓ tumor volume and multiplicity. **Effect**: Marine-based n-3 PUFA ↓ inflammatory markers more effectively than plant-based n-3 PUFA.
Liu et al., 2020 [65] USA	Female C57BL/6 mice	Induction by mouse E0771 cells (orthotopic) into 4th MG	Not specified	None	FO-HFD (45% fat) vs. HFD (45% fat) or LFD (10% fat) for 5 months (*n* = 11/group)	**Markers**: Tumor F4/80^+^CD11b^+^ macrophages, TAMs (F4/80^+^CD11b^+^Ly6C^−^MHCII^−^), A-FABP expression in macrophages **Results**: FO-induced obesity did not accelerate tumor growth, while HFD did. FO-HFD ↓ protumor TAMs in tumors and spleen and ↓ tumor weight and volume (*p* < 0.01). FO-HFD ↑ A-FABP in protumor TAMs. **Effect**: FO-HFD ↓ protumor macrophages.
Mandal et al., 2010 [74] USA	Immune-compromised nu/nu mice	Induction by MDA-MB-231 cells (intracardiac injection); TNBC	Not specified	None	10% FO (DHA + EPA) for 6 weeks vs. standard lab chow (*n* = 5/group)	**Markers**: Tumor CD44 protein and mRNA **Results**: FO ↓ expression of CD44 protein and mRNA (*p* < 0.05) **Effect**: FO ↓ metastasis via CD44.
Manni et al., 2011 [91] USA	Female FVB PyMT transgenic mice	Transgenic	3 weeks	Tamoxifen (1, 10, and 100 ppm)	FO (5%, 10% and 17%) + Tamoxifen for 11 weeks vs. 20% corn oil (CO; modified AIN-76 A) (*n* = 10/group for subset analyses)	**Markers**: Tumor PGF-2α, PPARα, PPARγ, PPARβ/δ, ADRP, Angptl4, AREG, FGB gene expression **Results**: FO dose-dependently ↓ PGF-2α (*p* < 0.05) due to reductions in tissue AA. ↔ in AREG, PPARα, PPARγ, PPARβ/δ, ADRP, Angptl4, FGB, or tumor parameters (multiplicity, volume or weight). **Effect**: FO ↓ AA-derived proinflammatory eicosanoid, PGF-2α, but this did not translate into further prevention of tumorigenesis.
Monk et al., 2021 [5] Canada	Female FVB MMTV-neu(ndl)-YD5 mice	Transgenic; HER-2+	4 weeks	None	Menhaden oil (FO) + HFD (corn oil + lard) for 16 weeks vs. LF or HFD (*n* = 10–12/group)	**Markers**: Tumor protein activation of NF-κB p65 and STAT3. Tumor mRNA expression of TNF-α, IL-6, IL-10, IL-1β, CCL2/MCP-1, PAI-1 leptin, resistin, and adiponectin **Results**: FO + HFD ↓ TNF-α, IL-6, leptin, PAI-1, resistin, NF-κB p65 and STAT3 and ↑ adiponectin and IL-10 (*p* < 0.05). **Effect**: FO ↓ proinflammatory transcription factors, cytokines and adipokines, and ↑ anti-inflammatory markers.
Negi et al., 2016 [13] India	Female Wistar rats (100–150 g)	Induction by drug DMBA (orally, single dose of 15 mg in 1 mL olive oil)	4 weeks	Celecoxib (20 mg/kg/BW)	0.5 mL Maxepa FO (180 mg EPA + 120 mg DHA) ± celecoxib for 7 d pretreatment followed by DMBA-induced carcinogenesis and 90 d of monitoring, vs. DMBA-only or control (olive oil vehicle) (*n* = 8/group)	**Markers**: Nuclear localization and expression of NF-κB p65 and COX-2; IFN-γ, IL-4, IL-10 in mammary tissue **Results**: FO ± celecoxib ↓ NF-κB p65, COX-2, IFN-γ, IL-4, and IL-10 compared with DMBA control (*p* < 0.05). **Effect**: FO ↓ inflammatory mediators.
Olivo et al., 2005 [61] USA	Female Sprague-Dawley rats	Induction by DMBA (oral gavage, 10 mg)	50 d	None	LF (*n* = 55) or HFD (*n* = 52; 20 mg DHA) n-3 PUFA (FO) from 5–25 d vs. LF (*n* = 52) or HFD (*n* = 25) n-6 PUFA; DMBA carcinogenesis at 50 d with 18 weeks of monitoring	**Markers**: COX-2 expression in MG, serum lipid peroxidation, apoptosis (TUNEL assay) and proliferation **Results**: LF n-3 PUFA ↓ COX-2, ↑ lipid peroxidation, ↑ apoptosis, ↓ proliferation in TEBs and LAUs and ↓ mammary tumor incidence (*p* < 0.05). HFD n-3 PUFA ↓ COX-2, ↑ lipid peroxidation, ↓ apoptosis, ↑ proliferation, and ↑ p-Akt and ↑ mammary tumor incidence (*p* < 0.05). **Effect**: LF n-3 PUFA contributed to ↓ inflammatory environment and was shown to be protective, whereas HFD n-3 PUFA ↑ mammary tumorigenesis.
Robinson et al., 2001 [62] Canada	Female Fischer 344 rats (~ 145 g)	Induction by R3230AC cells (subcutaneous injection of tumor brei in inguinal region)	Not specified	None	High n-3 (FO; 50 g LCFA/kg of fat) for 38 d (21 d pre- + 17 d post-implantation) vs. low n-3 diet (ALA; 0 g LCFA/kg) (*n* = 7–10/group)	**Markers**: CD5^+^,CD4^+^, CD8^+^T cells, CD25^+^, CD28^+^cells, B cells, macrophages, TNF-α, IFN-γ production in splenocytes **Results**: High n-3 diet ↑ IFN-γ, TNF-α, CD8^+^ and CD25^+^ CD8^+^ T cells; ↓ CD4:CD8 ratio (*p* < 0.05) compared to low n-3 diet. **Effect**: High n-3 diet ↑ immune cell activation and cytokine production.
Robinson et al., 2002 [63] Canada	Female Fischer 344 rats (~ 143 g)	Induction by R3230AC cells (subcutaneous injection of tumor brei in inguinal region)	Not specified	None	High (1.0) or low (0.35) PUFA: SFA ratios containing 20% w/w fat with high n-3 (FO; 5% w/w LCFA) or low n-3 (ALA; 0% w/w LCFA) for 38 days (*n* = 7–10/group)	**Markers**: NK cell cytotoxicity, splenocyte NO and IL-2 production, CD25^+^CD8^+^ and CD25^+^CD28^+^ cells **Results**: High n-3 diet (low PUFA: SFA ratio, 0.35) ↑ NK cell, NO, CD25^+^CD8^+^ and CD25^+^CD28^+^ cells in tumor-bearing rats, and ↑ IL-2 in healthy rats compared to the low n-3 diet (*p* < 0.05). ↔ in tumor growth or diets with high PUFA: SFA. **Effect**: High n-3 diet ↑ immune responses.
Silva et al., 2023 [92] Brazil	Female BALB/c mice	Induction by 4T1 tumor cells (injected into posterior left flank)	6–7 weeks	None	4% FO for 7 weeks vs. 4% linseed (ALA), canola or soybean (control) oils (*n* = 7/group)	**Markers**: Tumor MPO and NAG enzyme activity, TNF-α, VEGF and Hb content **Results**: FO ↑ MPO, ↓ NAG, ↑ MPO/NAG ratio and ↑ VEGF levels (*p* < 0.05). ↔ in TNF-α, Hb (vascular index), tumor weight or evolution. **Effect**: FO ↑ neutrophil recruitment and angiogenesis.
Souza et al., 2024 [50] Brazil	Female BALB/c mice (18–22 g)	Induction by 4T1 mammary carcinoma cell injection into the mammary fat pad (TNBC) on day 1	6–8 weeks	5-fluorouracil (5-FU; 300 mg/kg i.p.) to induce mucositis on day 17	FO (7% w/w total fat; 50% FO + 50% soy oil; 15% EPA and 7% DHA) vs. control (7% soy oil) supplementation began on day 10 and continued for 10 days post tumor inoculation (*n* = 10/group) for a total of 20 days study duration (10 days of FO supplementation)	**Markers**: Tumor IL-6, IL-1β, TNF-α, IL-10 (mRNA expression) **Results**: FO-fed mice ↓ tumor IL-6, IL-10 and TNF-α mRNA expression vs. control and after 5-FU treatment (*p* < 0.05). **Effect**: FO attenuated 5-FU associated inflammatory responses; ↓ tumor-associated inflammation by reducing IL-6 and TNF-α expression.
Turbitt et al., 2015 [64] USA	Female HER-2/neu FVB and PyMT FVB transgenic mice	Transgenic; HER-2/neu; PyMT	6–8 week HER-2/neu mice; 3 week PyMT mice	None	10% Menhaden oil (FO) for vs. corn oil (CO); HER-2/neu mice: *n* = 30/group; duration 16 or 50–65 weeks of age; PyMT mice: *n* = 10–12/group; duration 11 weeks of age	**Markers**: Tumor and splenic NK^+^ cells, F4/80^+^ / CD11b^+^ macrophages, Gr-1^+^ granulocytes, CD19^+^ B cells, CD3^+^/CD4^+^/CD8^+^T cells, CD11c^+^ dendritic cells, IL-2 and IFN-γ **Results**: FO ↑ IL-2 and IFN-γ production by T cells, ↓ tumor incidence and multiplicity, and ↑ tumor immune infiltrates (NK cells, F4/80^+^, and Gr-1^+^/ CD11b^+^ myeloid cells) in HER-2/neu mice (*p* < 0.05). FO ↑ splenic immune cells (NK cells, F4/80^+^, and Gr-1^+^/ CD11b^+^, CD4^+^, CD11c^+^, *p* < 0.05) in HER-2 and PyMT mice. **Effect**: FO ↑ antitumor immune responses.
Yun et al., 2016 [70] USA	Female *fat-1* transgenic mice and C57/BL6 WT control mice	Induction by E0771 cells (subcutaneous)	8–10 weeks	None	Endogenous formation of n-3 PUFA from n-6 PUFA (DHA-rich environment) via *fat-1* transgene vs. WT mice (control); duration 2–4 weeks post-tumor implantation	**Markers**: Xenograft tumor NF-κB activity, apoptosis (TUNEL assay), tumor growth and lung metastasis **Results**: *Fat-1* mice with DHA-rich environment ↓ tumor volume (–80%), NF-κB, apoptosis and metastasis (*p* < 0.05). **Effect**: DHA-rich environment ↓ inflammatory microenvironment, apoptosis and metastasis.
Zhu et al., 2011 [51] USA	Female Sprague Dawley rats	Induction by MNU (50 mg/kg BW, IP)	20 d	Tamoxifen (1 mg/kg diet)	n-3 PUFA (high n-3:n-6) for 8 weeks ± Tamoxifen vs. n-6 PUFA (low n-3:n-6) (*n* = 30/group)	**Markers**: Plasma leptin, adiponectin, IGF-I, CRP, IL-6, TNF-α levels **Result**: Higher n-3:n-6 ↓ leptin, IGF-I and ↑ adiponectin (*p* < 0.05). ↔ in CRP, IL-6, TNF-α with differing n-3:n-6 ratios. **Effect**: High n-3:n-6 modulated plasma hormones (leptin, adiponectin, IGF-I) but did not affect plasma cytokines (CRP, IL-6, TNF-α).
Zou et al., 2013 [59] France	Female *fat-1* transgenic mice and WT littermate controls	Induction by E0771 cells (subcutaneous injection into lower abdominal mammary fat pad)	10–12 weeks	None	Endogenous n-3 PUFA via *fat-1* transgene (*n* = 10) vs. WT mice (*n* = 6) fed high n-6 PUFA (safflower oil, SO diet) for 25 d	**Markers**: Tumor volume, tumor HER-2/HER3 signalling pathway activity, tumor lipid mediators (HEPE, HDHA, PG, TX), tumor NF-κB protein expression **Result**: *Fat-1* mice ↓ p-HER-3 (*p* < 0.01); ↓ expression of NF-κB, HER-2, HER-3, c-Myc in immunoblots (no p-value reported); ↓ PGD_2_, TXB_2_; ↑ n-3 derived PGE_3_, TXB_3_, DHA-(10-, 13-, 14-, 17-HDHA) derived mediators, EPA-(5-, 12-, 15-, 18-HEPE) derived mediators in tumors (*p* < 0.05). ). ↔ in PGE_2_. **Effect**: n-3 PUFA-rich environment ↑ anti-inflammatory lipid mediators and ↓ HER-2/HER-3 signaling.

↑, Higher than control/baseline; ↔, No difference from control/baseline; ↓, Lower than control/baseline. Unless otherwise specified

NF-κB is a transcription factor that regulates the expression of inflammatory cytokines, chemokines, and adhesion molecules and is activated by upstream signals including TNF-α signalling, IL-1β and toll-like receptor pathways [60]. COX-2 is an inducible enzyme that catalyzes AA metabolism to prostaglandins and other eicosanoids that promote tumor growth and inflammation [54, 55]. EPA and/or DHA supplementation from fish oil (equivalent to high-dose human supplementation of 3–4 g/day or amounts found in traditional Japanese diets) downregulated NF-κB activity and COX-2 expression [5, 9, 12, 13, 59], while one study observed no changes in NF-κB levels [52]. COX-2 expression was further found to be reduced in adjacent mammary gland tissue [61]. A study also noted a significant decrease in COX-2 and NF-κB expression after supplementation with 10% w/w flaxseed oil (ALA) from *in utero* to 20 weeks in a mouse model of human epidermal growth factor receptor 2-positive (HER-2+) breast cancer [9].

Supplementing with high levels of n-3 PUFA, including 3% fish oil in a 10% (w/w) fat diet (~ 1–2% of daily energy) or 2–4% DHA in a 20% (w/w) fat diet (~ 4–8% of daily energy), reduced pro-inflammatory tumor eicosanoids (e.g., PGE_2,_ 12-HETE and 15-HETE) and tumor Ki67 antigen expression, a cell proliferation marker [9, 57]. Studies also observed improvements in immune cell profiles, with n-3 PUFA increasing infiltration of cytotoxic T cells (CD8+) into splenocytes and natural killer (NK) cells into splenocytes and tumors, along with increasing associated markers in tumors (e.g., IL-2, IFN-γ and IL-10) [52, 62–64]. In parallel, n-3 PUFA decreased protumor tumor-associated macrophages (TAMs; F4/80 + CD11b+Ly6C−MHCII−) and total tumor F480 + cells, shifting macrophage polarization toward an anti-tumor phenotype [52, 65].

#### In Vitro Studies

The 30 in vitro studies consistently demonstrated that n-3 PUFA treatment led to significant reductions in inflammation-related signaling pathways in breast cancer cell lines **(**Table 3**)**. Decreases in NF-κB activation were reported in multiple studies utilizing the MCF-7 (ER+/PR+), MDA-231 (TNBC) and 4T1 (TNBC) cell lines [66–69]. Notably, treatment with DHA (5–50 µM) alone decreased NF-κB in MDA-MB-231 (TNBC) cells [28, 70]. Similarly, EPA (45–100 µM) was reported to decrease NF-κB, alongside downstream inflammatory markers, IL-6 and IL-8 mRNA expression, in both MDA-MB-231 cells and MCF-7 cells (*p* < 0.05) [28, 71].

COX-2 expression was significantly downregulated following treatment with n-3 PUFA EPA and DHA ethyl esters (EEs) (10–40 µM, 48 h) in MCF-7 (ER+/PR+) and MDA-231 (TNBC) cells [67]. Consistently, fish oil treatment (1 µL/mL; 63% n-3, 51% EPA + 21.4% DHA) reduced COX-2 expression in MCF-7 cells (ER+/PR+) [72]. In MDA-MB-231 cells (a TNBC cell line), EPA or DHA (50–200 µM), with or without genistein (0.1–2.5 µM, 24 h) decreased both PGE_2_ levels and COX-2 mRNA expression compared to AA treatment, while another study found that EPA or DHA (50 ng/mL, 21 h) treatment also reduced mRNA and protein expression of pro-metastatic marker CD44 [73, 74]. Separately, in the same cell line (MDA-MB-231), ALA treatment lowered secreted PGE_2_ levels and COX-2 mRNA, however, did not alter COX-2 protein expression [75]. Another study reported that DHA was further found to decrease PGE_2_ levels and COX-2 activity in BT-474 human breast ductal carcinoma cells (ER+/PR+/HER-2+) [76].

DHA treatment (5-200 µM) was found to significantly reduce matrix metalloproteinases (MMPs) that regulate inflammatory response and facilitate tumor progression [77, 78], including MMP-2, MMP-9 and MMP-11 transcription and/or activity in [66, 70, 76, 79, 80] in MDA-MD-231 (TNBC) and hormone receptor-positive MCF-7 (ER+) and BT-474 (ER+, PR+, HER-2+) cell lines. In one study, docosahexaenoic acid ethanolamide (DHEA), a derivative of DHA was also found to decrease MMP-9 expression in TAMs in MDA-MB-231 and MDA-MD-436 cells lines of TNBC [81]. In addition, DHA treatment (50–200 µM) was reported to dose-dependently increase heme oxygenase-1 (HO-1) in both MCF-7 (ER+) and MDA-MB-231 (TNBC) cells [66, 80], a rate-limiting enzyme of heme degradation that plays a role in reducing inflammation and tissue damage upon activation [82, 83]. Additionally, DHA (100 µM) significantly attenuated the hypoxia-induced overexpression of immune-checkpoint genes involved in tumor progression (PD-L1, CD80, CD39, CD47, CD73, B7-H3/CD276 and B7-H4) in MDA-MB-231 (TNBC) and BT-474 (ER+, PR+, HER-2+) cells [84].

**Table 3:** The characteristics of included studies investigating the effect of n-3 PUFA on biomarkers of inflammation in breast cancer: in vitro studies

Author, Year, Country	Cell line	Type of tumor	Concurrent treatment	Treatment vs. Control (Source/Dose/Duration)	Findings related to inflammation
Al-Jawadi et al., 2020 [69] USA	MCF-7 and MDA-MB-231	ER+/PR + and TNBC	None	EPA (100 µM) ± adipocyte (3T3-L1 or HMSC) CM (ACM) for 48 h vs. BSA + control medium or ACM	**Markers**: IL-6, NF-κB, STAT3 levels and mRNA transcription **Results**: EPA ↓ IL-6, NF-κB, NF-κB P-p65, STAT3, p-STAT3, cIAP2, FASN and mTOR in MDA-MB-231 cells exposed to control or ACM (*p* < 0.05). EPA ↓ NF-κB, NF-κB P-p65, STAT3, p-STAT3, cIAP2 and FASN in MCF-7 cells exposed to ACM (*p* < 0.05). ↔ in IκBα. **Effect**: EPA ↓ proinflammatory signalling, with greater effects in the more aggressive MDA-MB-231 cells.
Alquobaili et al., 2010 [93] USA	ER− (HCC-1806, MDA-MB-468, Hs578T and SK-BR-3) and ER+ (HCC-70, MCF-7, HCC-1500 and CAMA-1)	ER − and ER+	None	EPA (10 µM) for 6 and 24 h vs. control (no EPA)	**Markers**: HIF-1α, -3α, TGFα, IL-1R2 gene expression **Results**: EPA ↑ HIF-1α (+ 1.7), HIF-3α (+ 1.9) in ER+ cells, and ↓ TGFα (− 1.2), IL-1R2 (− 5.3) in ER− cells. **Effect**: EPA modulated inflammatory and hypoxia pathways, with receptor-specific anti-inflammatory and tumor-modulatory effects.
Altenburg et al., 2009 [94] USA	MDA-MB-231	TNBC	None	EPA and DHA (12.5, 25 and 50 µmol/L) for 24–48 h vs. controls (untreated, mock control or stearic acid)	**Markers**: CXCR4 surface and protein expression **Results**: EPA/DHA dose-dependently ↓ CXCR4 surface expression (− 75% with 50 µmol/L, *p* < 0.05). ↔ in CXCR4 effects between EPA and DHA. ↔ in total CXCR4 protein expression. EPA/DHA ↓ CXCR4-mediated migration toward CXCL12 (− 75–85% at 24 h, *p* < 0.05). **Effect**: EPA/DHA ↓ metastatic potential.
Altenburg et al., 2011 [95] USA	SK-BR-3	HER-2+	None	DHA (30 µM) or DHA (18 µM) ± curcumin (12 µM) for 24 h vs. untreated or vehicle control (ethanol or DMSO)	**Markers**: PPARγ protein, phospho-p53 activity, CXCL1, CXCR4, NLRP1 gene expression **Results**: DHA + curcumin ↑ pro-apoptotic and tumor-suppressive markers and ↓ metastatic and tumor-promoting markers, including ↑ PPARγ, phospho-p53, NLRP1; ↓ CXCL1, CXCR4, (*p* < 0.05). **Effect**: DHA alone did not alter tumour-related markers.
Augimeri et al., 2023 [81] Italy	MDA-MB-231 and MDA-MB-436, co-cultured with THP-1 monocytes	TNBC	None	DHEA (10 µM) for 24 h. vs. vehicle control/CM	**Markers**: TAMs recruitment, IL-6, IL10, CCL2/MCP-1, CCL5, MMP-9, VEGF gene expression, PPARγ activity and expression **Results**: DHEA ↓ CCL5, TAMs, IL-6, VEGF, CCL2/MCP-1, IL-10, and MMP-9 expression in TAMs, and ↑ PPARγ mRNA (*p* < 0.05). **Effect**: DHEA ↓ macrophage recruitment and proinflammatory markers.
Chénais et al., 2020 [80] France	MDA-MB-231	TNBC	None	100 µM DHA for 12–24 h vs. untreated cells	**Markers**: IL-1α, IL-1β, IL-12 A, IL-20Rβ, IL-24, HO-1, TRIB3, C5AR1, MMP11, TGFβ3, TNIP3, CXCL2, -3, FGFR3 gene expression **Results**: DHA ↑ IL-1α (+ 2.39), IL-1β (+ 1.55), IL-12 A (+ 1.12), IL-20Rβ (+ 2.03), IL-24 (+ 1.66), HO-1 (+ 2.17), TRIB3 (+ 2.22); C5AR1 (+ 3.96), CXCL2 (+ 1.64), CXCL3 (+ 1.72), TNIP3 (+ 1.01) and ↓ TGFβ3 (− 1.05), MMP-11 (− 1.02), FGFR3 (− 1.06), *p* < 0.05. **Effect**: DHA ↑ genes associated with anti-inflammatory responses.
Chen et al., 2013 [66] Taiwan	MCF-7	ER+/PR+	12-O-tetradecanoylphorbol-13-acetate (TPA, 100 ng/mL)	DHA (50–200 µM) or LA for 20 h prior to TPA + 24 h-post exposure vs. untreated or TPA-treated MCF-7 cells	**Markers**: MMP-9 (mRNA, protein, enzyme activity), ERK, PI3K/Akt, and PKC activation, NF-κB, AP-1 activity, HO-1 mRNA and protein expression **Results**: DHA ↓ TPA-induced MMP-9 mRNA, expression and activity via ↓ NF-κB/AP-1 signaling and dose-dependent ↑ HO-1 (*p* < 0.05). DHA ↓ ERK, PI3K/Akt, and PKC signaling (*p* < 0.05). **Effect**: DHA ↓ metastatic potential.
Chen et al., 2016 [67] USA	MCF-7, CAMA-1, MDA-MB-231, and SKBR3	ER+/PR+, TNBC, HER-2+	None	n-3 EEs (Lovaza; 10, 20, 40 µM) for 48 h vs. untreated cells	**Markers**: COX-2 (gene and protein expression), Bcl-2, PPARγ and NF-κB transcriptional activity, NF-κB p65 translocation **Results**: n-3 EEs ↓ COX-2, NF-κB activity and p65 translocation in MCF-7 and MDA-231 cells (*p* < 0.05). ↑ COX-2, NF-κB and p65 translocation in SKBR3 and ↔ in CAMA-1 cells. n-3 EEs ↓ PPARγ in SKBR3 only (*p* < 0.05). ↓ Bcl-2 in MCF-7, MDA-231 and CAMA-1 cells (*p* < 0.05). **Effect**: n-3 EEs ↓ NF-κB signaling and downstream targets independently of PPARγ in MCF-7 and MDA-MB-231 cells, showing selective anti-inflammatory and anti-tumor effects.
Chen et al., 2021 [56] Canada	BT-474, SK-BR-3, MDA-MB-468	ER+/PR+, HER-2+, TNBC	None	4-OXO-DHA (synthetic, 25 µM, 72 h exposure) vs. vehicle control (DMSO, 0.1%)	**Markers**: NF-κB and PPARɣ activity **Result**: 4-OXO-DHA ↓ pNF-κB and ↑ PPARɣ activity (AUC: BT-474, 57 ± 1; SK-BR-3, 124 ± 3; MDA-MB-468, 153 ± 2; all *p* < 0.05). **Effect**: 4-OXO-DHA exerted anti-inflammatory effect by ↓ NF-κB and ↑ PPARγ activity.
Chung et al., 2015 [85] USA	Py230 tumor cells derived from PyVmT/C57B mouse	TNBC	None	DHA (100 µM) for 1 h prior to treatment with vehicle, TNF-α (10 ng/ml) for 10 min or LPS (100 ng/ml) for 15 min	**Marker**: NF-κB activity **Result**: DHA ↓ NF-κB activation (p-TAK1 and p-IKK α/β) and nuclear translocation (NF-κB p65). **Effect**: DHA pre-treatment ↓ TNFα/LPS-induced inflammation.
Davison et al., 2018 [72] UK	MCF-7 and TamR	ER+/PR+	Signal transduction inhibitors: PD98059 (MEK1 inhibitor, 25 µM), LY294002 (PI3K inhibitor, 5 µM); 4-hydroxytamoxifen (4-OHT) for MCF-7 only	FO (1 µL/mL; 63% n-3, 51% EPA + 21.4% DHA) ± PD98059 + LY294002 for 48 h vs. DMSO (vehicle control)	**Markers**: COX-2 and p-Src protein expression **Results**: FO ↓ p-Src and COX-2 (*p* < 0.05). PD98059 + LY294002 also ↓ COX-2 to a lesser extent, but did not decrease p-Src. FO + PD98059 + LY294002 further decreased COX-2 and reduced p-Src expression. **Effect**: FO ↓ pro-inflammatory and metastatic/migration-related markers.
Dyari et al., 2017 [86] Australia	MDA-MB-231 cells	TNBC	None	C20E (40 µM) for 48 h vs. vehicle control	**Marker**: Caspase-3 and − 9 protein activity, Bax, Bak, Bcl-2, Bcl-xL protein expression **Results**: C20E ↑ caspase-3 and − 9, Bax, Bak, and ↓ Bcl-2 and Bcl-xL (*p* < 0.01). TRADD, TRAF1 and TNFR2 knockdown prevented the C20E-meadiated ↑ in cleaved caspase-3 (*p* < 0.001). **Effect**: C20E activated proapoptotic signaling via the TNFR1.
Ghaffari-Makhmalbaf et al., 2021 [96] Iran	MDA-MB-231 and BT-20	TNBC	None	DHA (50 µM) for 48 h vs. vehicle (ethanol)	**Markers**: VEGF mRNA expression and protein secretion, exosomal microRNAs (pro-angiogenic: miR-9, miR-17-5p, miR-19a, miR-126, miR-130a, miR-132, miR-296, miR-378; anti-angiogenic: miR-34a, miR-125b, miR-221, miR-222) expression **Results**: DHA ↓ VEGF-A mRNA and protein levels (*p* < 0.05), ↓ pro-angiogenic miRNAs (miR-9, miR-17-5p, miR-126), ↑ anti-angiogenic miRNAs (miR-34a, miR-125b) (*p* < 0.05). **Effect**: DHA ↓ angiogenesis via modulating VEGF and exosomal miRNAs.
Gionfriddo et al., 2020 [15] The Netherlands	MCF-7, MDA-MB-231, THP-1 monocytes	ER+/PR+, TNBC	PPARγ antagonist (GW9662)	DHEA (5 µM) and DHA-5-HT (1 µM) for 72 h vs. control (no treatment or PPARγ antagonist, GW9662)	**Markers**: PPARγ protein expression, IL-6 secreted protein levels in TAMs, IL-1Ra secreted protein levels in TAMs, CD80, CD206, CD163, CCL2/MCP-1, IL-1β, TNF-α gene expression in TAMs **Results**: DHA-5-HT ↓ IL-6 in TAMs co-cultured with MCF-7 and MDA-MB-231 CM. DHEA ↓ IL-1Ra in TAMs co-cultured with MCF-7 CM only (*p* < 0.05).Effects of DHEA and DHA-5-HT were reversed by PPARγ antagonist. ↔ in CD80, CD163 and CD206 macrophage surface markers. DHA-5-HT and DHEA ↓ TNF-α in TAMs of MCF-7 and MDA-MB-231 cells and ↓ IL-1β in MDA-MB-231 only (*p* < 0.05). **Effect**: DHA conjugates ↓ TAM cytokine secretion via PPARγ.
Hammamieh et al., 2007 [97] USA	MDA-MB-231, MDA-MB-435s, HCC2218, MCF-7	ER+/–, HER-2+, TNBC	None	EPA or DHA (10 µM) for 6 and 24 h vs. AA or LA (10 µM)	**Markers**: IL1RAP, CD84 gene expression **Results**: EPA and DHA ↓ IL1RAP and ↑ CD84 at 6 h compared to AA or LA n-6 PUFA (*p* < 0.05). **Effect**: n-3 PUFA ↓ proinflammatory signalling and ↑ immune response.
Hannafon et al., 2015 [98] USA	MCF-7 and MDA-MB-231, ZR-75-1, and BT20	ER+/PR+, TNBC	None	DHA (100 µM) for 24 h vs. vehicle control (ethanol)	**Markers**: Exosomal miRNAs (Let-7a, miR-23b, miR-27b, miR-21, miR-320b), PLAU, AMOTL1, NRP1, and ETS2 gene expression **Results**: DHA ↑ let-7a, miR-23b, miR-27b, miR-21, and miR-320b in BC exosomes. miRNAs from DHA-treated MCF-7 exosomes ↓ PLAU, AMOTL1, NRP1, and ETS2 in EA.hy926 endothelial cells. **Effect**: DHA ↑ anti-angiogenic miRNAs.
He et al., 2022 [68] The Netherlands	4T1 mouse mammary carcinoma	TNBC	None	DHA (10 µM) for 48 h vs. control (untreated)	**Markers**: CD86, CD68, CD163 protein expression (surface), IL-10, IL-12 secreted protein levels, NF-κB and STAT3, Snail mRNA expression, Ki67 protein expression **Results**: DHA ↓ NF-κB, STAT3, Snai1 and Ki-67. DHA ↔ macrophage polarization or cytokine secretion. **Effect**: DHA ↓ proliferation and inflammatory signalling, but did not affect immune cell polarization.
Horia et al., 2005 [75] USA	MDA-MB-231	TNBC	None	ALA or SDA (10–200 µM) vs. AA or BSA vehicle	**Markers**: COX-2 protein and mRNA expression, PGE_2_ secretion, NF-κB and PPARγ mRNA expression **Results**: ALA and SDA ↓ COX-2 mRNA compared to control and ↓ PGE_2_ compared to AA-treated cells (*p* < 0.05). ALA ↔ COX-2 protein levels or NF-κB and PPARγ mRNA. **Effect**: ALA and SDA ↓ COX-2 mRNA and PGE2, indicating an anti-inflammatory effect, but ↔ NF-κB or PPARγ mRNA levels.
Horia et al., 2007 [73] USA	MDA-MB-231	TNBC	Genistein (0.1–2.5 µM)	EPA or DHA (50–200 µM) ± genistein for 24 h vs. AA or control BSA and DMSO vehicle	**Markers**: COX-2, NF-κB and PPARγ mRNA expression, PGE_2_ secretion **Results**: EPA/DHA ± genistein ↓ PGE_2_ production compared to AA-treated cells, and ↓ COX-2 and NF-κB mRNA. EPA/DHA + genistein ↑ PPARγ (*p* < 0.05). **Effect**: EPA/DHA ↓ pro-inflammatory signalling.
Hwang et al., 2017 [99] China/Korea	MCF-7	ER+/PR+	12-O-tetradecanoylphorbol-13-acetate (TPA, 20 nM)	DHA (50 and 100 µM) for 24 h vs. vehicle control (DMSO)	**Markers**: MMP-9 mRNA and protein expression and activity, NF-κB (p50/p65) transcriptional activity, PPAR-γ protein expression, AP-1 transcriptional activity, MAPK protein phosphorylation/signalling activity (p-p38, p-ERK, p-JNK) **Results**: DHA dose-dependently ↓ TPA-induced MMP-9 expression and activity, ↓ p-p38, p-ERK; ↓ NF-κB and nuclear translocation of p50 and p65, ↑ PPAR-γ (*p* < 0.05). ↔ in AP-1 or p-JNK. **Effect**: DHA ↓ metastatic potential and proinflammatory signalling.
Javadian et al., 2020 [79] Iran	MDA-MB-231	TNBC	Taxol/Paclitaxel (0.3 µM)	DHA (100 µM) ± Taxol vs. vehicle control (ethanol/10% fatty acid-free BSA buffer)	**Markers**: MMP-2, -9, vimentin, HIF-1α, talin2 mRNA expression, miRNA (miR-194, miR0106b) **Results**: DHA and DHA + Taxol ↓ MMP-2, -9, vimentin, HIF-1α and ↑ talin2 and miR-194 (tumor suppressor), compared to control (*p* < 0.05). DHA + Taxol, but not DHA alone ↓ miR-106b (oncogenic) compared to control (*p* < 0.05). **Effect**: DHA ↓ metastatic potential.
Mandal et al., 2010 [74] USA	MDA-MB-231	TNBC	None	DHA or EPA (50 ng/mL) for 21 h for migration/invasion assay vs. untreated cells	**Markers**: CD44 expression **Results**: DHA and EPA ↓ expression and transcription of CD44. **Effect**: DHA and EPA ↓ pro-metastatic CD44 expression via transcription.
Maralbashi et al., 2024 [84] Iran	MDA-MB-231 and BT-474	TNBC, ER+/PR+/HER-2+	None	DHA (100 µM) for 24 h under normoxia or hypoxia (25 µM CoCl₂ for 24 h) vs. vehicle (BSA buffer)	**Markers**: CD39, CD73, CD47, CD80, PD-L1, B7-H3 (CD276), B7-H4, and regulatory miRNAs (miR-142-3p, miR-30a-5p, miR-133a, miR-194, miR-424, miR-155-5p, miR-143) (gene expression in cells and exosomes) **Results**: Hypoxia ↑ all immune-checkpoint genes, while DHA ↓ their mRNA expression in both cell lines (*p* < 0.0001); DHA ↑ tumor-suppressor miRNAs in both normoxic and hypoxic conditions across both cell lines (*p* < 0.05), with the exception of miR-133a in hypoxia; Largest ↓ for CD39 (-2674-fold change in BT-474; -21.5-fold change in MDA-MB-231 cells) and B7-H3 (-40.9 in BT-474 cells) in normoxia with DHA (*p* < 0.0001). **Effect**: DHA ↓ hypoxia-induced immunosuppressive and inflammatory signaling by reducing checkpoint gene and exosomal expression and enhancing anti-inflammatory miRNAs.
Mouradian et al., 2014 [76] USA	BT-474 human breast ductal carcinoma	ER+/PR+/HER-2+	None	DHA (100 µM) for 48 h vs. LA or ethanol	**Markers**: COX-2 activity, PGE_2_ secretion, TGF-α, MMP-2, MMP-9 protein expression **Results**: DHA ↓ COX-2 activity, PGE2 production, MMP-2 and MMP-9 compared to control or LA. DHA ↓ TGF-α compared to LA (*p* < 0.05). **Effect**: DHA ↓ inflammation-associated markers.
Newell et al., 2019 [100] Canada	MDA-MB-231	TNBC	Doxorubicin (DOX, 0.41 µM)	DHA (60 µM) ± DOX for 48 h vs. control (OALA, 40 µM LA + 40 µM oleic acid) ± DOX	**Markers**: Caspase-10, caspase-9, RIPK1, CXCL10, TRAF1 gene expression **Results**: DHA + DOX ↑ pro-apoptotic genes including caspase-10 (+ 1.27), caspase-9 (+ 1.35), RIPK1 (+ 1.21), CXCL10 (+ 2.69), TRAF1 (+ 1.43) compared to OALA + DOX control (*p* < 0.05). Compared with DOX alone, DHA + DOX increased caspase-10 (+ 1.16), caspase-9 (+ 1.17), CXCL10 (+ 1.87), and TRAF1 (+ 1.30), but not RIPK1. **Effect**: DHA alone did not increase pro-apoptotic/death receptor genes.
Pizato et al., 2018 [101] Brazil	MDA-MB-231 and 4T1	TNBC	None	DHA (50–200 µM) for 24 h vs. control (untreated) or AA	**Markers**: Caspase-1activation, gasdermin D cleavage, IL-1β secretion, HMGB1 and NF-κB protein translocation **Results**: DHA ↑ caspase-1, gasdermin D cleavage, IL-1β secretion, NF-κB translocation to nucleus, HMGB1 translocation to cytoplasm and membrane pore formation. **Effect**: DHA ↑ pyroptosis, pro-inflammatory programmed cell death.
Rasha et al., 2020 [71] USA	MDA-MB-231 and MCF-7	TNBC, ER+/PR+	Captopril (CAP, 100 µM) (Angiotensin-converting enzyme (ACE) inhibitor)	Direct EPA (100 µM) ± CAP or adipocyte-CM pre-treated with EPA ± CAP for 48 h vs. control (BSA CM or untreated)	**Markers**: NF-κB, STAT3, FASN mRNA expression, IL-6 and IL-8 mRNA expression and secretion **Results**: EPA and EPA + CAP-treated adipocyte-CM ↓ NF-κB, STAT3, FASN, IL-6 and IL-8 mRNA in MDA-MB-231 cells (*p* < 0.05). EPA-treated adipocyte-CM ↓ IL-6 and IL-8 secretion in MDA-MB-231 cells compared with untreated adipocyte-CM. Direct EPA and EPA + CAP ↓ IL-6 and IL-8 mRNA in MCF-7 cells (*p* < 0.05). **Effect**: EPA ↓ adipocyte-mediated proinflammatory markers and oncogenic signalling.
Schley et al., 2005 [28] Canada	MDA-MB-231	TNBC	None	EPA (60 µM) + DHA (40 µM) or EPA (45 µM) + DHA (30 µM) + LA (75 µM) for 72 h vs. LA (75 or 150 µM) or untreated cells	**Markers**: Caspase activation, NF-κB (p50 and p65) transcriptional activity, p-Akt (Ser473) activation, PCNA, PRK protein expression **Results**: EPA + DHA ± LA ↑ caspase activation compared to LA alone; ↓ PCNA, PRK, NF-κB (p50 and p65), p-Akt (*p* < 0.05). **Effect**: EPA + DHA ↑ apoptotic markers and ↓ tumor cell proliferation.
Wang et al., 2021 [102] Korea	MCF-7 and MDA-MB-231	ER+/PR+, TNBC	None	13R,20-diHDHA (20 and 40 µM) for 24–48 h vs. DMSO (vehicle control)	**Markers**: Cleaved-caspase 3 activation and protein expression, IL-6 secretion and protein expression, p-STAT3 activation, NF-κB p65 translocation, ROS activity, Nanog, Sox2, Oct4, c-Myc, and CD44 gene expression **Results**: 13R,20-diHDHA ↑ ROS; ↓ IL-6, p-STAT3, p65 in mammospheres and ↓ Nanog, Sox2, Oct4, c-Myc, and CD44 cancer stem cells (*p* < 0.05). ↔ in cleaved caspase-3. **Effect**: 13R,20-diHDHA ↓ oncogenic signalling.
Yun et al., 2016 [70] USA	MDA-MB-231 and T47D cells	TNBC and ER+	None	5–50 µM DHA (algae) for up to 24 h vs. AA (purity > 98%, all)	**Markers**: Cell proliferation, NF-κB, COX-2 protein expression and activity, β-catenin signaling, MMP-2 and MMP-9 transcription/activity, caspase-3 activity and PARP cleavage **Result**: DHA ↓ cell proliferation, NF-κB, COX-2, β-catenin signalling, and MMP-2/-9. AA ↑ MMP, but effects were blocked by DHA. DHA ↑ apoptosis via caspase-3 activation and PARP cleavage (*p* < 0.05). **Effect**: DHA ↓ pro-inflammatory and metastatic pathways and ↑ apoptosis.

↑, Higher than control/baseline; ↔, No difference from control/baseline; ↓, Lower than control/baseline. Unless otherwise specified

## Discussion

This systematic review evaluated the impact of n-3 PUFA in modulating breast cancer-associated inflammation, synthesizing evidence from human, animal, and in vitro studies. The overall evidence underscores the potential of n-3 PUFA to reduce inflammation and modulate the TME. Despite the anti-inflammatory and immunomodulatory effects observed in most studies, notable inconsistencies in some results, likely due to variability in study design, population, breast cancer stage or subtype, and/or doses of n-3 PUFA, highlight critical gaps in understanding n-3 PUFA-mediated mechanisms and clinical relevance.

The synthesis of prostaglandins (PGs) and pro-inflammatory and resolution-mediating eicosanoids is mediated by the cyclooxygenase (COX), lipoxygenase (LOX), and cytochrome P450 (CYP450) enzymes [103, 104], which catalyze the metabolism of n-3 and n-6 PUFA into bioactive lipid mediators. Lipid mediators derived from n-6 PUFA, such as PGs and leukotrienes (e.g., PGE_2_ and LTB4), predominantly exert pro-inflammatory effects, while those derived from n-3 PUFA (e.g., PGE_3_), promote inflammation resolution and tissue repair [105, 106]. COX-2 expression, a key driver of PGE_2_ production, was significantly downregulated by DHA and EPA in vitro, as well as in animal models [9, 12, 13, 67, 72, 73]. PGE_2_ is a potent pro-inflammatory eicosanoid and is implicated in tumor growth, metastasis, and immune suppression [107]. The reviewed studies demonstrated that EPA and DHA effectively reduced COX-2 expression and activity, alongside reductions in PGE_2_ levels in TNBC and hormone receptor-positive models, which highlights one mechanism through which n-3 PUFA have the potential to attenuate tumor-promoting inflammation [9, 12, 49, 57, 58, 67, 72, 73, 76]. Studies investigating the effects of ALA reported reductions in COX-2 mRNA expression without corresponding changes in protein expression [75], potentially suggesting limited efficacy of plant-derived n-3 PUFA compared to marine-derived n-3 PUFA to affect eicosanoid production, although further study is required.

The integration of n-3 PUFA into cellular membranes, resulting from increased intakes, not only shifts the balance of lipid mediator production toward anti-inflammatory profiles, but also modulates intracellular signaling pathways, such as activation of NF-κB [108]. NF-κB is a critical transcription factor regulating the production of pro-inflammatory cytokines including IL-6 and TNF-α, whereas IL-6 is also a known stimulator of CRP synthesis in the liver [108]. In the studies reviewed, n-3 PUFA supplementation, including EPA, DHA and ALA, consistently reduced NF-κB expression and activity across cell culture and animal models [5, 9, 12, 13, 28, 56, 66, 67, 69, 70, 73, 85]. Significant reductions in NF-κB were observed in MDA-MB-231 cells (TNBC) treated with DHA and EPA, with corresponding decreases in IL-6 and IL-8 mRNA expression [69, 71]. These findings support the role of n-3 PUFA in mitigating the inflammatory milieu of breast cancer by targeting upstream transcriptional regulators of pro-inflammatory cytokines.

Clinical studies also showed significant reductions in IL-6, with one trial reporting a 70% decrease in plasma IL-6 levels compared to baseline after 12 weeks of fish oil and evening primrose supplementation [36]. This robust response aligns with preclinical findings that demonstrated reductions in plasma and tumor TNF-α and PGE2 secreted levels and gene expression in animal models [5, 9, 48–50, 58]. These cytokines are central to the inflammatory milieu of the TME, and their modulation by n-3 PUFA suggests a direct impact on tumor-promoting inflammation. However, the variability in human study outcomes is notable. While significant reductions in inflammatory mediators were observed in pre- or postmenopausal women undergoing chemotherapy [36, 37, 42], studies with shorter supplementation durations or no concurrent treatment reported null or inconsistent effects [20, 39]. Reductions in circulating IL-6 levels were significant, whereas changes in CRP were inconsistent [36–38, 41, 42, 44]. CRP is an acute-phase protein and a general marker of systemic inflammation, which may potentially be less sensitive to localized effects of n-3 PUFA within the TME [109, 110]. However, interpreting these findings are complicated by methodological differences across studies, including heterogeneity in patient populations such as breast cancer stage and type, wide age ranges and baseline inflammatory status, which may explain some of the inconsistencies in observed effects.

MMPs, including MMP-2 and MMP-9 are a class of zinc-dependent proteins that are critical mediators of extracellular matrix remodeling and metastasis [111]. MMP-2 and MMP-9 protein expression have been reported to be highly expressed in breast cancer tissues compared to adjacent normal tissues [112] and this overexpression is associated with poor overall prognosis, including greater lymph node metastasis, higher histological grades and larger tumor sizes [113]. In the present systematic review, DHA treatment was found to consistently suppress MMP activity and transcription in TNBC, ER + and HER-2 + breast cancer cell lines [66, 70, 76, 79, 80]. Additional reductions in MMP-9 expression were also observed in tumor-associated macrophages (TAMs) treated with DHEA in MDA-MB-231 and MDA-MD-436 cells [81]. DHEA is a DHA-derived endocannabinoid that has been found to be more effective than DHA in inhibiting breast cancer cell growth in MDA-MD-231 and MCF-7 cells, demonstrating potent anti-proliferative effects [27]. In the present review, DHEA was also found to decrease TAMs recruitment via decreased CCL2/MCP-1 and CCL5 chemokine expression, and reduce pro-inflammatory cytokine gene expression including IL-6 [81]. Similarly, another study reported that DHEA decreased gene expression of TNF-α and IL-1β in TAMs incubated with MDA-MD-231 cell conditioned media [15]. This study also assessed the effects of docosahexaenoyl serotonin (DHA-5-HT) on inflammatory markers, reporting reductions in IL-6 secreted protein levels and TNF-α gene expression levels in TAMs incubated with MCF-7 or MDA-MD-231 cell conditioned media [15]. DHA-5-HT is a DHA serotonin conjugate that possesses anti-inflammatory properties including downregulation of cytokines, chemokines (CCL2/MCP-1, CCL20) and MMPs gene expression in RAW264.7 macrophages [114].

Macrophages, including TAMs, are crucial components of the TME and play a significant role in shaping the tumor immune landscape. TAMs often exhibit an M2-like phenotype in breast cancer, which promotes tumor progression by secreting pro-inflammatory cytokines, angiogenic factors including VEGF, and matrix remodeling enzymes such as MMPs that facilitate metastasis [115]. The ability of DHA and its downstream derivatives (i.e., DHEA and DHA-5-HT) to reprogram TAMs toward a less inflammatory or anti-tumoral phenotype highlights an important mechanism by which n-3 PUFA may exert their anti-cancer effects. By reducing TAM-associated gene expression and/or secretion of inflammatory cytokines (e.g., IL-6, TNF-α) and chemokines (e.g., CCL2/MCP-1, CCL5), n-3 PUFA may decrease tumor inflammation and alter immune cell recruitment to the tumor site [9, 15, 81, 85]. Furthermore, the observed reductions in MMP activity and expression [70, 81] suggest that n-3 PUFA and their derivatives (i.e., DHEA) can limit metastatic progression by targeting both tumor cells and the surrounding stromal environment. These results underscore the immunomodulatory potential of n-3 PUFA in the TME and highlight the need for future studies to include markers of tumor infiltrating immune cells beyond TAMs and identify both their phenotype and function.

### Study Heterogeneity and Barriers to Clinical Translation

A major challenge in evaluating the anti-inflammatory potential of n-3 PUFA in breast cancer and translating the findings of this review into clinical practice lies in the considerable heterogeneity in study designs across in vitro, animal, and human studies. In vitro experiments differed in the type of cell lines used (e.g., ER+/PR+, HER-2+, and TNBC cells), exposure durations (ranging from 1 to 72 h), concentrations of n-3 PUFA assessed (ranging from 5 to 200 µM) [70, 101], and co-treatment conditions (e.g., with adipocyte-conditioned media, chemotherapeutics, or anti-inflammatory agents) [71], as described in Table 3. Moreover, the endpoints assessed across studies varied widely, from classic inflammatory cytokines and transcriptional factors (e.g., IL-6, TNF-α, NF-κB) to markers of metastasis, apoptosis, and emerging targets such as exosomal miRNAs. This variability was expected, as the review aimed to capture both well-established and novel markers of inflammation relevant to breast cancer. While this breadth provides valuable insights into the diverse mechanisms by which n-3 PUFA may exert anti-inflammatory effects in mammary tumors, it also introduces challenges in making direct cross-study comparisons. Animal studies were equally diverse (Table 2), encompassing transgenic, xenograft, and chemically-induced models across different mouse and rat strains [9, 52]. n-3 PUFA interventions varied by source (e.g., algae, fish oil, DHA, EPA), mode of delivery (gavage vs. dietary), dosage and duration [56, 61, 85]. Some studies incorporated concurrent treatments (e.g., chemotherapy, anti-inflammatory agents, Avastin, Tamoxifen) [13, 41, 43, 45, 51, 91], while others assessed maternal vs. postnatal exposure [9, 90]. Tumor subtypes also differed, including HER-2+, hormone receptor-positive (HR+), and TNBC. Endpoints were overall diverse and included immune cell infiltration, angiogenesis, lipid mediator and cytokine production.

Translating these preclinical findings to humans introduces further complexity due to interspecies differences in metabolism, immune responses, and tumor microenvironment dynamics. The 13 human clinical trials reviewed herein (Table 1) varied widely in their study designs, including differences in n-3 PUFA formulations, with some using purified algal-derived DHA [20], high-dose EPA + DHA triglycerides [37], or complex blends with co-supplemented bioactives such as gamma linolenic acid (GLA), curcumin, or hydroxytyrosol [36, 42]. Dosing regimens ranged from 1 g/day [46] to over 4 g/day [37, 40], with varying EPA: DHA ratios, sources (algal vs. marine), and molecular forms (triglycerides, ethyl esters). Intervention durations also differed across human studies, from short-term protocols of 30 days [39, 42] to longer 24-week duration interventions [37, 44]. Additionally, patient populations were diverse, encompassing women with different breast cancer subtypes and stages of disease (e.g., early-stage HR + vs. metastatic HER-2-negative, HER-2–), age (ranging from 18 to 84 years), menopause status (pre- vs. post-menopausal), obesity status (normal/unspecified vs. overweight/obese) treatment contexts (chemotherapy, endocrine therapy, or none), and baseline inflammatory or nutritional status. Some trials included treatment-naïve patients [39], while others focused on those undergoing active therapy [36, 38, 46] or in long-term survivorship [20], further limiting comparability. This variability likely contributed to the inconsistent findings across trials, with some reporting reductions in circulating IL-6 or CRP [36–38, 42], and others showing no significant anti-inflammatory effects despite comparable or higher dosing [39, 40, 44]. To improve comparability and enhance clinical translation, future trials should aim to standardize intervention protocols, report precise nutrient compositions, and stratify participants by breast cancer subtype, treatment status, and inflammatory or dietary profiles.

### Emerging Inflammation-Related Markers

This review highlighted several emerging inflammatory markers that warrant further investigation. While traditional markers such as IL-6, TNF-α, and CRP were consistently evaluated, novel markers including Lef1, Lta, HO-1, exosomal miRNAs, TRIB3, C5AR1, TGFβ3, and TNIP3 have gained attention for their potential roles in regulating inflammation in the TME [66, 80, 84, 90]. Emerging evidence suggests that exosomal miRNAs and proteins such as TRIB3 and C5AR1 could mediate macrophage-driven inflammatory responses and contribute to the pro-tumorigenic or anti-tumorigenic roles of the TME [116]. In the present review, DHA (50–100 µM) was found to downregulate oncogenic and pro-angiogenic exosomal miRNAs (i.e., miR-106b, miR-9, miR-17-5p and miR-126) and upregulate tumor-supressing and anti-angiogenic miRNAs (i.e.miR-194, miR-34a, miR-125b) in MDA-MD-231 cells (TNBC) [79, 96]. This result was accompanied with a decrease in VEGF-A mRNA and protein levels, suggesting that DHA decreases angiogenesis via modulating VEGF and exosomal miRNAs [96]. TRIB3 is a pseudokinase involved in various cellular and physiological functions, and has been linked to inflammation and cancer progression, as both an oncogene and tumor suppressor [117, 118]. In one study, TRIB3 gene expression was upregulated with DHA treatment (100 µM) in MDA-MB-231 cells (TNBC), accompanied by an increase in other genes that may play an anti-inflammatory role (i.e., IL-20Rβ and IL-24), suggesting that n-3 PUFA may modulate TRIB3 to influence inflammatory pathways associated with tumor growth inhibition [80]. C5AR1 plays a role in inflammatory responses and immune cell recruitment. Its upregulation with DHA treatment in MDA-MD-231 cells (TNBC) indicates that n-3 PUFA may disrupt pro-tumor inflammatory signaling mediated by the complement system [80].

Additionally, markers such as TGFβ3 and TNIP3, known for their regulatory roles in fibrosis and immune signaling, may serve as novel targets for exploring the broader anti-inflammatory mechanisms of n-3 PUFA. One study found that DHA (100 µM) downregulated TGFβ3, a member of the transforming growth factor-beta family, and upregulated TNIP3 within TNBC (MDA-MD-231 cells) [80]. Interestingly, elevated TGFβ3 expression is associated with breast tumorigenesis and lymph node metastasis [119]. Other reviewed studies also reported a decrease in TGFα gene or protein expression with EPA (10 µM) or DHA (100 µM) treatment, and a decrease in TGFβ and TGFβR2 protein expression with fish oil (containing 5.1–10.7 mg/g EPA and 3.7–8.3 mg/g DHA) supplementation across preclinical models of breast cancer (ER+/PR+, HER-2 + and TNBC) [76, 88, 93]. TNIP3 regulates NF-κB signaling and inflammation [120]. Its upregulation with DHA treatment in TNBC cells suggests that n-3 PUFA may suppress NF-κB-driven inflammatory pathways, thereby reducing tumor-promoting inflammation [80]. HO-1 is an enzyme with anti-inflammatory and antioxidant properties. Its upregulation has been associated with reduced inflammation and tumor growth, making it a potential target for n-3 PUFA modulation [121]. HO-1 gene expression was upregulated with DHA treatment in TNBC (MDA-MB-231 cells) and ER+/PR+ (MCF-7 cells) indicating that n-3 PUFA may enhance HO-1-mediated anti-inflammatory and antioxidant effects to inhibit tumor progression [66, 80].

Interestingly, maternal fish oil supplementation was found to increase Lef1 expression in C3(1) Tag offspring, a transgenic model that develops hormone-independent mammary tumors in the mammary epithelium driven by SV40 large T-antigen (Tag)-mediated inactivation of p53 and Rb, suggesting a potential regulatory role of n-3 PUFA in Wnt/β-catenin signaling [90, 122]. This aligns with growing evidence that the maternal diet can influence offspring disease susceptibility through epigenetic fetal programming and highlights how early-life exposure to n-3 PUFA may shape gene expression patterns and the risk of developing chronic disease later in life [123–125]. Lef1 is a transcription factor that is integral to immune cell development and inflammatory responses [126]. In breast cancer, Lef1 has been reported to be overexpressed, and hepatocyte growth factor (HGF) has been shown to upregulate Lef1 via Akt/NF-κB signaling, promoting migration and invasion of MDA-MB-231 cells [127, 128]. Beyond these tumor-intrinsic effects, Lef1 expression was found to be positively correlated with tumor-infiltrating immune cells, including macrophages, suggesting a link to an immune-modulatory role within the breast TME [129]. In non-cancer systems, miR-223 and miR-34a were shown to downregulate Lef1 expression, enabling B-cell-to-macrophage conversion, suggesting that reducing Lef1 can attenuate macrophage reprogramming [127, 130]. However, Lef1 may have context-dependent roles in cancer, as one study in colorectal cancer demonstrated that Lef1 loss increased tumor initiation and tumor cell proliferation by disrupting intestinal crypt formation, highlighting a tumor suppressive role [131]. Whether Lef1 plays a similar role in breast cancer remains unclear. Given the complex interplay between Wnt/β-catenin signaling, immune modulation, and tumor development [132], the implications of the observed upregulation of Lef1 expression following n-3 PUFA supplementation require further investigation to determine whether Lef1 plays a pro-tumorigenic or tumor-suppressive role in breast cancer-associated inflammation.

Lipid mediators such as PGE_2_, HETEs and RvD1/2 also emerged as critical players. Animal studies demonstrated reductions of up to 90% in tumor PGE_2_ levels alongside decreases in pro-inflammatory mediators including 12- and 15-HETE with n-3 PUFA supplementation [57, 58]. In parallel, increases in anti-inflammatory eicosanoids, including EPA-derived HEPEs, DHA-derived HDHAs and PGE_3_, were observed in animal studies [59]. In humans, DHA supplementation (2 g/day) was also reported to elevate plasma RvD1 and RvD2 levels in BRCA1/2-mutated patients [47]. These mediators are actively involved as intermediates in signalling pathways that resolve inflammation and promote tissue homeostasis, suggesting that n-3 PUFA do not only suppress inflammation but actively facilitate its resolution [133]. Therefore, the anti-inflammatory actions of n-3 PUFA may be mediated by their bioactive derivatives to suppression tumorigenesis.

### Implications and Future Directions

The findings of this systematic review underscore the potential of n-3 PUFA as adjuvant therapies for modulating inflammation in breast cancer. However, critical gaps remain that warrant further investigation to optimize clinical application. One primary challenge in translating these findings into clinical practice is the variability in study designs, including differences in n-3 PUFA formulations, doses, intervention durations, and patient populations. Future clinical trials should aim for standardization by implementing well-defined dosing regimens and stratifying patients based on breast cancer subtypes, inflammatory status, age and baseline dietary patterns. Harmonizing these study design elements is essential to identify responsive subgroups for robust comparisons, support reproducibility, and clarify the therapeutic potential of specific n-3 PUFA formulations in breast cancer. Additionally, further research is needed to elucidate the relative potency and efficacy of individual fatty acids in breast cancer prevention.

Risk of bias across the included studies also highlights important areas for improvement. Almost half of the human clinical trials assessed were judged to have a high overall risk of bias according to the RoB 2 assessment. Although some studies demonstrated strengths across multiple domains, isolated methodological shortcomings, such as inadequate randomization procedures, missing allocation concealment, insufficient blinding, or incomplete outcome data handling, often contributed to high overall risk ratings under the RoB 2 framework. Selective reporting, which was frequently linked to the absence of pre-registered protocols, was another common concern. These limitations may reduce confidence in the reported findings and underscore the need for greater methodological rigor in future research. To improve research quality and mitigate sources of bias, future trials should adopt robust randomization methods, ensure proper allocation concealment, maintain blinding where feasible, and adhere to comprehensive protocol pre-registration and transparent reporting. Addressing these methodological shortcomings will be essential to strengthen the evidence base and support the clinical translation of n-3 PUFA interventions.

Future studies should also investigate how n-3 PUFA modulate emerging inflammatory markers in different breast cancer subtypes and their potential implications for tumor progression and metastasis. The role of n-3 PUFA in regulating macrophage polarization and immune cell recruitment also warrants further mechanistic studies, particularly in the context of immunotherapy. Evidence suggests that n-3 PUFA may enhance the efficacy of chemotherapy and immunotherapy by modulating the TME, offering a promising avenue for integrated therapeutic strategies [134–137]. Combining n-3 PUFA with immune checkpoint inhibitors or other immunomodulatory agents may provide synergistic effects that enhance anti-tumor immunity [134].

The interplay between lipid mediators and the broader inflammatory signaling network remains an important area of research. While this review highlights the ability of n-3 PUFA to reduce PGE_2_ and COX-2 expression, additional studies are needed to fully characterize the spectrum of bioactive lipid mediators involved in breast cancer inflammation. Advances in lipidomics and single-cell spatial profiling and transcriptomics could provide deeper insights into the specific inflammatory pathways influenced by n-3 PUFA.

Finally, long-term studies with extended follow-up periods are needed to evaluate the sustained impact of n-3 PUFA supplementation on breast cancer outcomes, including recurrence and survival. While preclinical evidence strongly supports their anti-inflammatory and tumor-suppressive effects, robust epidemiological and interventional studies are essential to establish causality and guide clinical recommendations. Additionally, given the accessibility and safety profile of n-3 PUFA, their incorporation into dietary guidelines and public health strategies for breast cancer prevention should be explored.

### Strengths and Limitations

Strengths of this systematic review were the comprehensive search strategy, inclusion of various study types and a wide range of inflammatory and immune-related outcomes. This approach enabled the thorough synthesis of findings across preclinical and clinical studies to identify key areas for future research, including emerging inflammatory markers and different bioactive forms of n-3 PUFA, and provide considerations for clinical practice. Despite these strengths, some limitations warrant consideration. The heterogeneity in the reported study outcomes and methodologies, including variations in n-3 PUFA formulations, doses, intervention durations, and patient populations, limited direct comparisons between studies. As a result, a meta-analysis was not feasible. Also, while the overall evidence supports the role of n-3 PUFA supplementation in modulating inflammation in breast cancer, the lack of standardized dosing strategies across clinical trials makes it challenging to establish optimal therapeutic recommendations. Further research is needed to elucidate the specific forms and effective doses of n-3 PUFA and their potential synergistic interactions with other anti-cancer agents.

## Conclusion

This systematic review underscores the potential of n-3 PUFA to modulate inflammation and the TME in breast cancer, with robust evidence from preclinical studies and promising, findings in human trials. Targeting the precise mechanisms by which n-3 PUFA exert their anti-inflammatory effects and understanding the full scope of their immunomodulatory properties in breast cancer is an important area of research. However, challenges remain in translating these findings into clinical practice. Addressing methodological variability and leveraging emerging biomarkers will be critical to optimizing the therapeutic potential of n-3 PUFA in breast cancer treatment and management. Further, clinical trials investigating the long-term effects of n-3 PUFA supplementation on breast cancer outcomes and inflammatory markers are warranted to help establish their potential as a therapeutic intervention.

## Key References

- Bray F, Laversanne M, Sung H, Ferlay J, Siegel RL, Soerjomataram I, et al. Global cancer statistics 2022: GLOBOCAN estimates of incidence and mortality worldwide for 36 cancers in 185 countries. CA Cancer J Clin. 2024;74(3):229–63. 10.3322/caac.21834.
  - ○ Provides up-to-date global cancer burden with standardized methods, framing the public-health significance of breast cancer and prevention strategies.
- Guo Y, Ma B, Li X, Hui H, Zhou Y, Li N, et al. n-3 PUFA can reduce IL-6 and TNF levels in patients with cancer. British Journal of Nutrition. 2023;129(1):54–65. 10.1017/S0007114522000575.
  - ○ Meta-analysis of RCTs linking n-3 PUFA to reductions in key circulating chronic inflammatory cytokines in different patient populations, supporting biologic plausibility for anti-inflammatory effects of n-3 PUFA in breast cancer.
- Jamali M, Zarezadeh M, Jamilian P, Ghoreishi Z. The effect of n-3 polyunsaturated fatty acids on inflammation status in cancer patients: Updated systematic review and dose- and time-response meta-analysis. PharmaNutrition. 2024;27:100372. 10.1016/j.phanu.2023.100372.
  - ○ Quantifies dose and duration relationships, guiding practical trial design and clinical dosing considerations for anti-inflammatory endpoints across all cancers.
- Sahye-Pudaruth S, Ma DWL. Assessing the Highest Level of Evidence from Randomized Controlled Trials in Omega-3 Research. Nutrients. 2023;15(4):1001.
  - ○ Offers a pragmatic framework for judging RCT quality and heterogeneity in n-3 PUFA trials, directly informing interpretation of clinical evidence summarized in this review.
- Theinel MH, Nucci MP, Alves AH, Dias OFM, Mamani JB, Garrigós MM, et al. The Effects of Omega-3 Polyunsaturated Fatty Acids on Breast Cancer as a Preventive Measure or as an Adjunct to Conventional Treatments. Nutrients. 2023;15(6):1310.
  - ○ Comprehensive update on preventative and adjuvant roles of n-3 PUFA across experimental animal models, situating the field’s current consensus and gaps.
- Munhoz J, Bigras G, Newell M, Rivas-Serna IM, Mazurak V, Goruk S, et al. The effects of docosahexaenoic acid (DHA) on plasma cytokines, oxylipins, and tumor-infiltrating lymphocytes from women with breast cancer undergoing neoadjuvant chemotherapy in the DHA-WIN trial. The Journal of Nutritional Biochemistry. 2025;145:110025. 10.1016/j.jnutbio.2025.110025.
  - ○ Prospective clinical data showing DHA-linked modulation of circulating oxylipins and tumor-immune features alongside chemotherapy, bridging mechanistic lipid mediators to the tumor microenvironment.
- Almassri HF, Abdul Kadir A, Srour M, Foo LH. The effects of Omega-3 fatty acids and vitamin D supplementation on the quality of life and blood inflammation markers in newly diagnosed breast cancer women: An open-labelled randomised controlled trial. Clinical Nutrition ESPEN. 2025;65:64–75. 10.1016/j.clnesp.2024.11.014.
  - ○ Highlights early clinical signals that combined nutrient supplementation may improve quality-of-life and inflammatory markers during initial treatment; underscores need for confirmatory blinded trials.
- Marchio V, Augimeri G, Morelli C, Vivacqua A, Giordano C, Catalano S, et al. Omega-3 fatty acids: molecular weapons against chemoresistance in breast cancer. Cellular & Molecular Biology Letters. 2025;30(1):11. 10.1186/s11658-025-00694-x.
  - ○ Synthesizes emerging mechanisms by which n-3 PUFA may overcome chemoresistance (membrane remodeling, signaling, metabolism reprogramming), highlighting translational opportunities.

## Supplementary Information

Below is the link to the electronic supplementary material.

Supplementary Material 1 (DOCX 17.3 KB)

## Data Availability

No datasets were generated or analysed during the current study.

## Abbreviations

(AUDA): 12-(3-adamantan-1-yl-ureido)-dodecanoic acid
(5-LOX): 5-lipoxygenase
(DMBA): 7,12-Dimethylbenz(a)anthracene
(AP-1): Activator Protein-1
(ADRP): Adipose Differentiation-Related Protein
(ALA): alpha-linolenic acid
(AREG): Amphiregulin
(AMOTL1): Angiomotin like-1
(Angptl4): Angiopoietin-like Protein 4
(Ki-67): Antigen Kiel 67
(AA): Arachidonic acid
(AI): Aromatase inhibitor
(B7-H3): B7 Homolog 3
(B7-H4): B7 Homolog 4
(BW): Body weight
(BSA): Bovine serum albumin
(BC): Breast cancer
(BI-RADS): Breast Imaging-Reporting and Data System
(CCL): C-C motif ligand
(CRP): C-reactive protein
(Cer): Ceramide
(CXCL): Chemokine(C-X-C motif) ligand
(CD84): Cluster of Differentiation 84
(C5AR1): Complement C5a Receptor 1
(CLS-B): Crown-like structures of the breast
(DCIS): Ductal carcinoma in situ
(DHA): Docosahexaenoic acid
(DHEA): Docosahexaenoic acid ethanolamide
(EPA): Eicosapentaenoic acid
(EAC): Ehrlich ascites carcinoma
(EGF): Epidermal growth factor
(EREG): Epiregulin
(ER): Estrogen receptor
(EEs): Ethyl esters
(ERK): Extracellular Signal-Regulated Kinase
(FASN): Fatty acid synthase
(FGB): Fibrinogen beta chain
(FGFR3): Fibroblast Growth Factor Receptor 3
(FGFR): Fibroblast growth factor
(FO): Fish oil
(GPR120): G-protein-coupled receptor 120
(GLA): Gamma linolenic acid
(G-CSF): Granulocyte colony-stimulating factor
(GM-CSF): Granulocyte-macrophage colony-stimulating factor
(HO-1): Heme Oxygenase 1
(Hb): Hemoglobin
(HGF): Hepatocyte growth factor
(HMGB1): High mobility group box 1
(hsCRP): High sensitivity C-reactive protein
(HR+): Hormone receptor-positive
(HER-2): Human epidermal growth factor receptor 2
(IDC): Invasive Ductal Carcinoma
(IFNγ): Interferon gamma
(IL): Interleukin
(IL1RAP): Interleukin-1 receptor accessory protein
(IGF-1): Insulin-like Growth Factor-1
(IP): Intraperitoneal
(IV): Intravenous
(LTB4): Leukotriene B4
(LA): Linoleic acid
(LPS): Lipopolysaccharide
(LCIS): Lobular carcinoma in situ
(LAUs): Lobulo-alveolar units
(Lymphotoxin A): Lta
(Lef1): Lymphoid enhancer binding factor 1
(MIP): Macrophage inflammatory protein
(MDA): Malondialdehyde
(MG): Mammary gland
(MMTV): Mammary Tumor Virus
(MMPs): Matrix metalloproteinases
(miRNAs): MicroRNAs
(MCP-1): Monocyte chemoattractant protein-1
(MPO): Myeloperoxidase
(NAG): N-acetylglucosaminidase
(DHA-5-HT): N-docosahexaenoyl serotonin
(NK): Natural killer
(NE): Neutrophil elastase
(NLR): Neutrophil to T-lymphocyte ratio
(NRP1): Neuropilin 1
(MNU): N-methyl-N-nitrosourea
(NO): Nitric oxide
(NLRP1): NOD-like receptor pyrin domain-containing protein 1
(OVX): Ovariectomized
(PA): Palmitic Acid
(PBMC): Peripheral blood mononuclear cells
(PPARα): Peroxisome proliferator-activated receptor alpha
(PPARβ/δ): Peroxisome proliferator-activated receptor beta/delta
(PPARγ): Peroxisome proliferator-activated receptor gamma
(PHA): Phytohemagglutinin
(PAI-1): Plasminogen Activator Inhibitor-1
(PLAU): Plasminogen activator
(PyMT): Polyoma middle T
(PD-L1): Programmed Death-Ligand 1
(PCNA): Proliferating Cell Nuclear Antigen
(PRK): Proliferation-related Kinase
(PR): Progesterone receptor
(PGF-2α): Prostaglandin F2-alpha
(PG): Prostaglandin
(PKC): Protein Kinase C
(ROS): Reactive oxygen species
(RvD1): Resolvin D1
(RvD2): Resolvin D2
(Se): Selenium
(SAA): Serum amyloid A
(SPMs): Specialized pro-resolving mediators
(SM): Sphingomyelin
(SDA): Stearidonic acid
(Th): T helper cell
(Treg): T regulatory cell
(TEBs): Terminal end buds
(TGFα): Transforming growth factor alpha
(TGFβ): Transforming growth factor beta
(TNIP3): TNFAIP3 interacting protein 3
(TRIB3): Tribbles Pseudokinase 3
(TG): Triglyceride
(TEM): Tumor microenvironment
(TNF-α): Tumor necrosis factor alpha
(TNFR1): Tumor necrosis factor receptor 1
(TNFR2): Tumor necrosis factor receptor 2
(TRADD): Tumor necrosis factor receptor type 1-associated death domain protein
(TAMs): Tumor-associated macrophages
(ETS2): V-ets erythroblastosis virus E26 oncogene homolog 2
(VEGF): Vascular endothelial growth factor
(WAT): White adipose tissue
(WT): Wild-type
(C20E): ω-3 17,18-epoxyeicosatetraenoic acid

## References

[1] Bray F, Laversanne M, Sung H, Ferlay J, Siegel RL, Soerjomataram I, et al. Global cancer statistics 2022: GLOBOCAN estimates of incidence and mortality worldwide for 36 cancers in 185 countries. CA Cancer J Clin. 2024;74(3):229–63. 10.3322/caac.21834. 38572751 10.3322/caac.21834

[2] Chen S, Cao Z, Prettner K, Kuhn M, Yang J, Jiao L, et al. Estimates and Projections of the Global Economic Cost of 29 Cancers in 204 Countries and Territories From 2020 to 2050. JAMA Oncol. 2023;9(4):465–72. 10.1001/jamaoncol.2022.7826. 36821107 10.1001/jamaoncol.2022.7826 PMC9951101

[3] Łukasiewicz S, Czeczelewski M, Forma A, Baj J, Sitarz R, Stanisławek A. Breast Cancer-Epidemiology, Risk Factors, Classification, Prognostic Markers, and Current Treatment Strategies-An Updated Review. Cancers (Basel). 2021;13(17). 10.3390/cancers13174287. 10.3390/cancers13174287 PMC8428369 34503097

[4] Greten FR, Grivennikov SI. Inflammation and Cancer: Triggers, Mechanisms, and Consequences. Immunity. 2019;51(1):27–41. 10.1016/j.immuni.2019.06.025. 31315034 10.1016/j.immuni.2019.06.025 PMC6831096

[5] Monk JM, Liddle DM, Hutchinson AL, Burns JL, Wellings H, Cartwright NM, et al. Fish oil supplementation increases expression of mammary tumor apoptosis mediators and reduces inflammation in an obesity-associated HER-2 breast cancer model. J Nutr Biochem. 2021;95:10. 10.1016/j.jnutbio.2021.108763. 10.1016/j.jnutbio.2021.108763 33965532

[6] Hillyer L, Hucik B, Baracuhy E, Lin Z, Muller W, Robinson L, et al. Her-2 Breast Cancer Outcomes Are Mitigated by Consuming n-3 Polyunsaturated, Saturated, and Monounsaturated Fatty Acids Compared to n-6 Polyunsaturated Fatty Acids. Nutrients. 2020;12:3901. 10.3390/nu12123901. 33419361 10.3390/nu12123901 PMC7766940

[7] Leslie MA, Abdelmagid SA, Perez K, Muller WJ, Ma DW. Mammary tumour development is dose-dependently inhibited by n-3 polyunsaturated fatty acids in the MMTV-neu(ndl)-YD5 transgenic mouse model. Lipids Health Dis. 2014;13:96. 10.1186/1476-511X-13-96. 24916956 10.1186/1476-511X-13-96 PMC4064525

[8] Simopoulos AP. Omega-3 fatty acids in inflammation and autoimmune diseases. J Am Coll Nutr. 2002;21(6):495–505. 10.1080/07315724.2002.10719248. 12480795 10.1080/07315724.2002.10719248

[9] Liu J, Abdelmagid SA, Pinelli CJ, Monk JM, Liddle DM, Hillyer LM, et al. Marine fish oil is more potent than plant-based n-3 polyunsaturated fatty acids in the prevention of mammary tumors. J Nutr Biochem. 2018;55:41–52. 10.1016/j.jnutbio.2017.12.011. 29413488 10.1016/j.jnutbio.2017.12.011

[10] Gabbs M, Leng S, Devassy JG, Monirujjaman M, Aukema HM. Advances in Our Understanding of Oxylipins Derived from Dietary PUFAs. Adv Nutr. 2015;6(5):513–40. 10.3945/an.114.007732. 26374175 10.3945/an.114.007732 PMC4561827

[11] Julliard WA, Myo YPA, Perelas A, Jackson PD, Thatcher TH, Sime PJ. Specialized pro-resolving mediators as modulators of immune responses. Semin Immunol. 2022;59:101605. 10.1016/j.smim.2022.101605. 35660338 10.1016/j.smim.2022.101605 PMC9962762

[12] Ford NA, Rossi EL, Barnett K, Yang PY, Bowers LW, Hidaka BH, et al. Omega-3-Acid Ethyl Esters Block the Protumorigenic Effects of Obesity in Mouse Models of Postmenopausal Basal-like and Claudin-Low Breast Cancer. Cancer Prev Res. 2015;8(9):796–806. 10.1158/1940-6207.capr-15-0018. 10.1158/1940-6207.CAPR-15-0018 PMC5024781 26100521

[13] Negi AK, Renuka, Bhatnagar A, Agnihotri N. Celecoxib and fish oil: a combination strategy for decreased inflammatory mediators in early stages of experimental mammary cancer. Inflammopharmacology. 2016;24(1):11–22. 10.1007/s10787-015-0259-7. 26749133 10.1007/s10787-015-0259-7

[14] Calder PC. n-3 PUFA and inflammation: from membrane to nucleus and from bench to bedside. Proc Nutr Soc. 2020;79(4):404–16. 10.1017/S0029665120007077. 10.1017/S0029665120007077 32624016

[15] Gionfriddo G, Plastina P, Augimeri G, Catalano S, Giordano C, Barone I, et al. Modulating Tumor-Associated Macrophage Polarization by Synthetic and Natural PPARγ Ligands as a Potential Target in Breast Cancer. Cells. 2020;9(1). 10.3390/cells9010174. 10.3390/cells9010174 PMC7017381 31936729

[16] Guo Y, Ma B, Li X, Hui H, Zhou Y, Li N, et al. n-3 PUFA can reduce IL-6 and TNF levels in patients with cancer. Br J Nutr. 2023;129(1):54–65. 10.1017/S0007114522000575. 35249562 10.1017/S0007114522000575

[17] Kavyani Z, Musazadeh V, Fathi S, Hossein Faghfouri A, Dehghan P, Sarmadi B. Efficacy of the omega-3 fatty acids supplementation on inflammatory biomarkers: An umbrella meta-analysis. Int Immunopharmacol. 2022;111:109104. 10.1016/j.intimp.2022.109104. 35914448 10.1016/j.intimp.2022.109104

[18] Jamali M, Zarezadeh M, Jamilian P, Ghoreishi Z. The effect of n-3 polyunsaturated fatty acids on inflammation status in cancer patients: Updated systematic review and dose- and time-response meta-analysis. PharmaNutrition. 2024;27:100372. 10.1016/j.phanu.2023.100372.

[19] Pan L, Zhou Y, Yin H, Hui H, Guo Y, Xie X. Omega-3 Polyunsaturated Fatty Acids Can Reduce C-Reactive Protein in Patients with Cancer: A Systematic Review and Meta-Analysis of Randomized Controlled Trials. Nutr Cancer. 2022;74(3):840–51. 10.1080/01635581.2021.1931365. 34060403 10.1080/01635581.2021.1931365

[20] Gucalp A, Zhou XK, Cook ED, Garber JE, Crew KD, Nangia JR, et al. A Randomized Multicenter Phase II Study of Docosahexaenoic Acid in Patients with a History of Breast Cancer, Premalignant Lesions, or Benign Breast Disease. Cancer Prev Res. 2018;11(4):203–13. 10.1158/1940-6207.capr-17-0354. 10.1158/1940-6207.CAPR-17-0354 PMC6290902 29453232

[21] Sahye-Pudaruth S, Ma DWL. Assessing the Highest Level of Evidence from Randomized Controlled Trials in Omega-3 Research. Nutrients. 2023;15(4):1001. 36839358 10.3390/nu15041001 PMC9959429

[22] Turner KM, Yeo SK, Holm TM, Shaughnessy E, Guan JL. Heterogeneity within molecular subtypes of breast cancer. Am J Physiol Cell Physiol. 2021;321(2):C343–54. 10.1152/ajpcell.00109.2021. 34191627 10.1152/ajpcell.00109.2021 PMC8424677

[23] Iacobuzio-Donahue CA, Litchfield K, Swanton C. Intratumor heterogeneity reflects clinical disease course. Nat Cancer. 2020;1(1):3–6. 10.1038/s43018-019-0002-1. 35121835 10.1038/s43018-019-0002-1

[24] Lüönd F, Tiede S, Christofori G. Breast cancer as an example of tumour heterogeneity and tumour cell plasticity during malignant progression. Br J Cancer. 2021;125(2):164–75. 10.1038/s41416-021-01328-7. 33824479 10.1038/s41416-021-01328-7 PMC8292450

[25] Hassan M, Tutar L, Sari-Ak D, Rasul A, Basheer E, Tutar Y. Non-genetic heterogeneity and immune subtyping in breast cancer: Implications for immunotherapy and targeted therapeutics. Transl Oncol. 2024;47:102055. 10.1016/j.tranon.2024.102055. 39002207 10.1016/j.tranon.2024.102055 PMC11299575

[26] Pogash TJ, El-Bayoumy K, Amin S, Gowda K, de Cicco RL, Barton M, et al. Oxidized derivative of docosahexaenoic acid preferentially inhibit cell proliferation in triple negative over luminal breast cancer cells. Vitro Cell Dev Biol Anim. 2015;51(2):121–7. 10.1007/s11626-014-9822-6. 10.1007/s11626-014-9822-6 PMC4948656 25413005

[27] Brown I, Lee J, Sneddon AA, Cascio MG, Pertwee RG, Wahle KWJ, et al. Anticancer effects of n-3 EPA and DHA and their endocannabinoid derivatives on breast cancer cell growth and invasion. Prostaglandins Leukot Essent Fat Acids. 2020;156:102024. 10.1016/j.plefa.2019.102024. 10.1016/j.plefa.2019.102024 31679810

[28] Schley PD, Jijon HB, Robinson LE, Field CJ. Mechanisms of omega-3 fatty acid-induced growth inhibition in MDA-MB-231 human breast cancer cells. Breast Cancer Res Treat. 2005;92(2):187–95. 10.1007/s10549-005-2415-z. 15986129 10.1007/s10549-005-2415-z

[29] Das UN. Tumoricidal action of cis-unsaturated fatty acids and their relationship to free radicals and lipid peroxidation. Cancer Lett. 1991;56(3):235–43. 10.1016/0304-3835(91)90008-6. 1850658 10.1016/0304-3835(91)90008-6

[30] VanderSluis L, Mazurak VC, Damaraju S, Field CJ. Determination of the Relative Efficacy of Eicosapentaenoic Acid and Docosahexaenoic Acid for Anti-Cancer Effects in Human Breast Cancer Models. Int J Mol Sci. 2017. 10.3390/ijms18122607. 29207553 10.3390/ijms18122607 PMC5751210

[31] Newell M, Mazurak V, Postovit LM, Field CJ. N-3 Long-Chain Polyunsaturated Fatty Acids, Eicosapentaenoic and Docosahexaenoic Acid, and the Role of Supplementation during Cancer Treatment: A Scoping Review of Current Clinical Evidence. Cancers (Basel). 2021;13(6). 10.3390/cancers13061206. 10.3390/cancers13061206 PMC8000768 33801979

[32] Theinel MH, Nucci MP, Alves AH, Dias OFM, Mamani JB, Garrigós MM, et al. The Effects of Omega-3 Polyunsaturated Fatty Acids on Breast Cancer as a Preventive Measure or as an Adjunct to Conventional Treatments. Nutrients. 2023;15(6):1310. 36986040 10.3390/nu15061310 PMC10052714

[33] Page MJ, McKenzie JE, Bossuyt PM, Boutron I, Hoffmann TC, Mulrow CD, et al. The PRISMA 2020 statement: an updated guideline for reporting systematic reviews. BMJ. 2021;372:n71. 10.1136/bmj.n71. 33782057 10.1136/bmj.n71 PMC8005924

[34] Bernasconi AA, Wilkin AM, Roke K, Ismail A. Development of a novel database to review and assess the clinical effects of EPA and DHA omega-3 fatty acids. Prostaglandins Leukot Essent Fatty Acids. 2022;183:102458. 10.1016/j.plefa.2022.102458. 35816925 10.1016/j.plefa.2022.102458

[35] Sterne JAC, Savović J, Page MJ, Elbers RG, Blencowe NS, Boutron I, et al. RoB 2: a revised tool for assessing risk of bias in randomised trials. BMJ. 2019;366:l4898. 10.1136/bmj.l4898. 31462531 10.1136/bmj.l4898

[36] Arsic A, Krstic P, Paunovic M, Nedovic J, Jakovljevic V, Vucic V. Anti-inflammatory effect of combining fish oil and evening primrose oil supplementation on breast cancer patients undergoing chemotherapy: a randomized placebo-controlled trial. Sci Rep. 2023;13(1):9. 10.1038/s41598-023-28411-8. 37081029 10.1038/s41598-023-28411-8 PMC10119093

[37] Lustberg MB, Orchard TS, Reinbolt R, Andridge R, Pan XL, Belury M, et al. Randomized placebo-controlled pilot trial of omega 3 fatty acids for prevention of aromatase inhibitor-induced musculoskeletal pain. Breast Cancer Res Treat. 2018;167(3):709–18. 10.1007/s10549-017-4559-z. 29101597 10.1007/s10549-017-4559-z PMC5809189

[38] Mantha OL, Hankard R, Tea I, Schiphorst AM, Dumas JF, Berger V et al. N-3 Fatty Acid Supplementation Impacts Protein Metabolism Faster Than it Lowers Proinflammatory Cytokines in Advanced Breast Cancer Patients: Natural <SUP>15</SUP> N/<SUP>14</SUP> N Variations during a Clinical Trial. Metabolites. 2022;12(10):19. 10.3390/metabo12100899 10.3390/metabo12100899 PMC9609900 36295801

[39] Paixao EMD, Oliveira ACD, Pizato N, Muniz-Junqueira MI, Magalhaes KG, Nakano EY, et al. The effects of EPA and DHA enriched fish oil on nutritional and immunological markers of treatment naive breast cancer patients: a randomized double-blind controlled trial. Nutr J. 2017;16:11. 10.1186/s12937-017-0295-9. 29061183 10.1186/s12937-017-0295-9 PMC5653994

[40] Hutchins-Wiese HL, Picho K, Watkins BA, Li Y, Tannenbaum S, Claffey K, et al. High-dose eicosapentaenoic acid and docosahexaenoic acid supplementation reduces bone resorption in postmenopausal breast cancer survivors on aromatase inhibitors: a pilot study. Nutr Cancer. 2014;66(1):68–76. 10.1080/01635581.2014.847964. 24274259 10.1080/01635581.2014.847964

[41] Munhoz J, Bigras G, Newell M, Rivas-Serna IM, Mazurak V, Goruk S, et al. The effects of docosahexaenoic acid (DHA) on plasma cytokines, oxylipins, and tumor-infiltrating lymphocytes from women with breast cancer undergoing neoadjuvant chemotherapy in the DHA-WIN trial. J Nutr Biochem. 2025;145:110025. 10.1016/j.jnutbio.2025.110025. 40651709 10.1016/j.jnutbio.2025.110025

[42] Martínez N, Herrera M, Frías L, Provencio M, Pérez-Carrión R, Díaz V, et al. A combination of hydroxytyrosol, omega-3 fatty acids and curcumin improves pain and inflammation among early stage breast cancer patients receiving adjuvant hormonal therapy: results of a pilot study. Clin Transl Oncol. 2019;21(4):489–98. 10.1007/s12094-018-1950-0. 30293230 10.1007/s12094-018-1950-0

[43] Almassri HF, Abdul Kadir A, Srour M, Foo LH. The effects of Omega-3 fatty acids and vitamin D supplementation on the quality of life and blood inflammation markers in newly diagnosed breast cancer women: An open-labelled randomised controlled trial. Clin Nutr ESPEN. 2025;65:64–75. 10.1016/j.clnesp.2024.11.014. 39577691 10.1016/j.clnesp.2024.11.014

[44] Hershman DL, Unger JM, Crew KD, Awad D, Dakhil SR, Gralow J, et al. Randomized Multicenter Placebo-Controlled Trial of Omega-3 Fatty Acids for the Control of Aromatase Inhibitor-Induced Musculoskeletal Pain: SWOG S0927. J Clin Oncol. 2015;33(17):1910–7. 10.1200/jco.2014.59.5595. 25940724 10.1200/JCO.2014.59.5595 PMC4451174

[45] Munhoz J, Newell M, Goruk S, Ghosh S, Patel D, Joy AA, et al. Docosahexaenoic acid (DHA) supplementation attenuates changes in the concentration, phenotype, and response of immune peripheral blood cells in breast cancer patients undergoing neoadjuvant therapy. Secondary findings from the DHA-WIN trial. Breast Cancer Res. 2025;27(1):91. 10.1186/s13058-025-02048-z. 40405290 10.1186/s13058-025-02048-z PMC12100857

[46] Darwito D, Dharmana E, Riwanto I, Budijitno S, Suwardjo S, Purnomo J, et al. Effects of Omega-3 Supplementation on Ki-67 and VEGF Expression Levels and Clinical Outcomes of Locally Advanced Breast Cancer Patients Treated with Neoadjuvant CAF Chemotherapy: A Randomized Controlled Trial Report. Asian Pac J Cancer Prev. 2019;20(3):911–6. 10.31557/apjcp.2019.20.3.911. 30912414 10.31557/APJCP.2019.20.3.911 PMC6825781

[47] Molfino A, Imbimbo G, Salerno G, Lionetto L, De Luca A, Simmaco M, et al. Effects of DHA Oral Supplementation on Plasma Resolvin D1 and D2 Levels in Naïve Breast Cancer Patients. Cancers. 2025. 10.3390/cancers17101694. 40427191 10.3390/cancers17101694 PMC12109739

[48] Guo CH, Hsia SM, Chung CH, Lin YC, Shih MY, Chen PC, et al. Nutritional supplements in combination with chemotherapy or targeted therapy reduces tumor progression in mice bearing triple-negative breast cancer. J Nutr Biochem. 2021;87:12. 10.1016/j.jnutbio.2020.108504. 10.1016/j.jnutbio.2020.108504 32956826

[49] Elisia I, Yeung M, Wong J, Kowalski S, Larsen M, Shyp T, et al. A low-carbohydrate diet containing soy protein and fish oil reduces breast but not prostate cancer in C3(1)/Tag mice. Carcinogenesis. 2022;43(2):115–25. 10.1093/carcin/bgab106. 34958345 10.1093/carcin/bgab106

[50] Souza ALA, Martins Trindade L, Dias Borges A, Caroline Lacerda Leocadio P, de Oliveira Silva J, Salgado Fernandes R, et al. Omega-3 fatty acid supplementation attenuates intestinal mucositis and tumor growth in a murine model of breast cancer. J Funct Foods. 2024;115:106096. 10.1016/j.jff.2024.106096.

[51] Zhu Z, Jiang W, McGinley JN, Prokopczyk B, Richie JP Jr., Bayoumy E. Mammary gland density predicts the cancer inhibitory activity of the N-3 to N-6 ratio of dietary fat. Cancer Prev Res (Phila). 2011;4(10):1675–85. 10.1158/1940-6207.capr-11-0175. 21813405 10.1158/1940-6207.CAPR-11-0175

[52] Khadge S, Thiele GM, Sharp JG, McGuire TR, Klassen LW, Black PN, et al. Long-chain omega-3 polyunsaturated fatty acids decrease mammary tumor growth, multiorgan metastasis and enhance survival. Clin Exp Metastasis. 2018;35(8):797–818. 10.1007/s10585-018-9941-7. 30327985 10.1007/s10585-018-9941-7

[53] Goupille C, Vibet S, Frank PG, Mahéo K. EPA and DHA Fatty Acids Induce a Remodeling of Tumor Vasculature and Potentiate Docetaxel Activity. Int J Mol Sci. 2020;21(14). 10.3390/ijms21144965. 10.3390/ijms21144965 PMC7404030 32674321

[54] Greenhough A, Smartt HJ, Moore AE, Roberts HR, Williams AC, Paraskeva C, et al. The COX-2/PGE2 pathway: key roles in the hallmarks of cancer and adaptation to the tumour microenvironment. Carcinogenesis. 2009;30(3):377–86. 10.1093/carcin/bgp014. 19136477 10.1093/carcin/bgp014

[55] Calder PC, Eicosanoids. Essays Biochem. 2020;64(3):423–41. 10.1042/ebc20190083. 32808658 10.1042/EBC20190083

[56] Chen KM, Thompson H, Vanden-Heuvel JP, Sun YW, Trushin N, Aliaga C, et al. Lipoxygenase catalyzed metabolites derived from docosahexaenoic acid are promising antitumor agents against breast cancer. Sci Rep. 2021;11(1):410. 10.1038/s41598-020-79716-x. 33431978 10.1038/s41598-020-79716-x PMC7801725

[57] Connolly JM, Gilhooly EM, Rose DP. Effects of reduced dietary linoleic acid intake, alone or combined with an algal source of docosahexaenoic acid, on MDA-MB-231 breast cancer cell growth and apoptosis in nude mice. Nutr Cancer. 1999;35(1):44–9. 10.1207/s1532791444-49. 10624705 10.1207/S1532791444-49

[58] Colombo DT, Tran LK, Speck JJ, Reitz RC. Comparison of hexadecylphosphocholine with fish oil as an antitumor agent. J Lipid Mediat Cell Signal. 1997;17(1):47–63. 10.1016/s0929-7855(97)00020-5. 9302654 10.1016/s0929-7855(97)00020-5

[59] Zou Z, Bellenger S, Massey KA, Nicolaou A, Geissler A, Bidu C, et al. Inhibition of the HER2 pathway by n-3 polyunsaturated fatty acids prevents breast cancer in fat-1 transgenic mice. J Lipid Res. 2013;54(12):3453–63. 10.1194/jlr.M042754. 24052576 10.1194/jlr.M042754 PMC3826691

[60] Liu T, Zhang L, Joo D, Sun S-C. NF-κB signaling in inflammation. Signal Transduct Target Therapy. 2017;2(1):17023. 10.1038/sigtrans.2017.23. 10.1038/sigtrans.2017.23 PMC5661633 29158945

[61] Olivo SE, Hilakivi-Clarke L. Opposing effects of prepubertal low- and high-fat n-3 polyunsaturated fatty acid diets on rat mammary tumorigenesis. Carcinogenesis. 2005;26(9):1563–72. 10.1093/carcin/bgi118. 15888492 10.1093/carcin/bgi118

[62] Robinson LE, Clandinin MT, Field CJ. R3230AC rat mammary tumor and dietary long-chain (n-3) fatty acids change immune cell composition and function during mitogen activation. J Nutr. 2001;131(7):2021–7. 10.1093/jn/131.7.2021. 11435524 10.1093/jn/131.7.2021

[63] Robinson LE, Clandinin MT, Field CJ. The role of dietary long-chain n-3 fatty acids in anti-cancer immune defense and R3230AC mammary tumor growth in rats: influence of diet fat composition. Breast Cancer Res Treat. 2002;73(2):145–60. 10.1023/a:1015261111605. 12088117 10.1023/a:1015261111605

[64] Turbitt WJ, Black AJ, Collins SD, Meng HC, Xu HF, Washington S, et al. Fish Oil Enhances T Cell Function and Tumor Infiltration and Is Correlated With a Cancer Prevention Effect in HER-2/neu But Not PyMT Transgenic Mice. Nutr Cancer. 2015;67(6):965–75. 10.1080/01635581.2015.1060351. 26226376 10.1080/01635581.2015.1060351

[65] Liu L, Jin R, Hao J, Zeng J, Yin D, Yi Y, et al. Consumption of the Fish Oil High-Fat Diet Uncouples Obesity and Mammary Tumor Growth through Induction of Reactive Oxygen Species in Protumor Macrophages. Cancer Res. 2020;80(12):2564–74. 10.1158/0008-5472.can-19-3184. 32213543 10.1158/0008-5472.CAN-19-3184 PMC7299802

[66] Chen HW, Chao CY, Lin LL, Lu CY, Liu KL, Lii CK, et al. Inhibition of matrix metalloproteinase-9 expression by docosahexaenoic acid mediated by heme oxygenase 1 in 12-O-tetradecanoylphorbol-13-acetate-induced MCF-7 human breast cancer cells. Arch Toxicol. 2013;87(5):857–69. 10.1007/s00204-012-1003-3. 23288142 10.1007/s00204-012-1003-3

[67] Chen CH, Fabian C, Hursting S, deGraffenried LA. Breast Cancer Genetic and Molecular Subtype Impacts Response to Omega-3 Fatty Acid Ethyl Esters. Nutr Cancer. 2016;68(6):1021–33. 10.1080/01635581.2016.1192199. 27367296 10.1080/01635581.2016.1192199 PMC12879510

[68] He Y, Rezaei S, Júnior RFA, Cruz LJ, Eich C. Multifunctional Role of Lipids in Modulating the Tumorigenic Properties of 4T1 Breast Cancer Cells. Int J Mol Sci. 2022;23(8):4240. 10.3390/ijms23084240 PMC9024985 35457057

[69] Al-Jawadi A, Rasha F, Ramalingam L, Alhaj S, Moussa H, Gollahon L, et al. Protective effects of eicosapentaenoic acid in adipocyte-breast cancer cell cross talk. J Nutr Biochem. 2020;75:8. 10.1016/j.jnutbio.2019.108244. 10.1016/j.jnutbio.2019.108244 31704550

[70] Yun EJ, Song KS, Shin S, Kim S, Heo JY, Kweon GR, et al. Docosahexaenoic acid suppresses breast cancer cell metastasis by targeting matrix-metalloproteinases. Oncotarget. 2016;7(31):49961–71. 10.18632/oncotarget.10266. 27363023 10.18632/oncotarget.10266 PMC5226561

[71] Rasha F, Kahathuduwa C, Ramalingam L, Hernandez A, Moussa H, Moustaid-Moussa N. Combined Effects of Eicosapentaenoic Acid and Adipocyte Renin-Angiotensin System Inhibition on Breast Cancer Cell Inflammation and Migration. Cancers. 2020;12(1):17. 10.3390/cancers12010220. 10.3390/cancers12010220 PMC7016836 31963198

[72] Davison Z, Nicholson RI, Hiscox S, Heard CM. Co-Administration of Fish Oil With Signal Transduction Inhibitors Has Anti-Migration Effects in Breast Cancer Cell Lines, in vitro. Open Biochem J. 2018;12:130–48. 10.2174/1874091x01812010130. 30288178 10.2174/1874091X01812010130 PMC6142674

[73] Horia E, Watkins BA. Complementary actions of docosahexaenoic acid and genistein on COX-2, PGE2 and invasiveness in MDA-MB-231 breast cancer cells. Carcinogenesis. 2007;28(4):809–15. 10.1093/carcin/bgl183. 17052999 10.1093/carcin/bgl183

[74] Mandal CC, Ghosh-Choudhury T, Yoneda T, Choudhury GG, Ghosh-Choudhury N. Fish oil prevents breast cancer cell metastasis to bone. Biochem Biophys Res Commun. 2010;402(4):602–7. 10.1016/j.bbrc.2010.10.063. 20971068 10.1016/j.bbrc.2010.10.063 PMC2993881

[75] Horia E, Watkins BA. Comparison of stearidonic acid and alpha-linolenic acid on PGE2 production and COX-2 protein levels in MDA-MB-231 breast cancer cell cultures. J Nutr Biochem. 2005;16(3):184–92. 10.1016/j.jnutbio.2004.11.001. 15741054 10.1016/j.jnutbio.2004.11.001

[76] Mouradian M, Kikawa KD, Johnson ED, Beck KL, Pardini RS. Key roles for GRB2-associated-binding protein 1, phosphatidylinositol-3-kinase, cyclooxygenase 2, prostaglandin E2 and transforming growth factor alpha in linoleic acid-induced upregulation of lung and breast cancer cell growth. Prostaglandins Leukot Essent Fat Acids. 2014;90(4):105–15. 10.1016/j.plefa.2013.12.001. 10.1016/j.plefa.2013.12.001 PMC4138981 24374147

[77] Hojilla CV, Wood GA, Khokha R. Inflammation and breast cancer. Metalloproteinases as common effectors of inflammation and extracellular matrix breakdown in breast cancer. Breast Cancer Res. 2008;10(2):205. 10.1186/bcr1980. 18394187 10.1186/bcr1980 PMC2397522

[78] Kessenbrock K, Plaks V, Werb Z. Matrix metalloproteinases: regulators of the tumor microenvironment. Cell. 2010;141(1):52–67. 10.1016/j.cell.2010.03.015. 20371345 10.1016/j.cell.2010.03.015 PMC2862057

[79] Javadian M, Shekari N, Soltani-Zangbar MS, Mohammadi A, Mansoori B, Maralbashi S, et al. Docosahexaenoic acid suppresses migration of triple-negative breast cancer cell through targeting metastasis-related genes and microRNA under normoxic and hypoxic conditions. J Cell Biochem. 2020;121(3):2416–27. 10.1002/jcb.29464. 31713924 10.1002/jcb.29464

[80] Chénais B, Cornec M, Dumont S, Marchand J, Blanckaert V. Transcriptomic Response of Breast Cancer Cells MDA-MB-231 to Docosahexaenoic Acid: Downregulation of Lipid and Cholesterol Metabolism Genes and Upregulation of Genes of the Pro-Apoptotic ER-Stress Pathway. Int J Environ Res Public Health. 2020;17(10). 10.3390/ijerph17103746. 10.3390/ijerph17103746 PMC7277693 32466294

[81] Augimeri G, Fiorillo M, Morelli C, Panza S, Giordano C, Barone I, et al. The Omega-3 Docosahexaenoyl Ethanolamide Reduces CCL5 Secretion in Triple Negative Breast Cancer Cells Affecting Tumor Progression and Macrophage Recruitment. Cancers (Basel). 2023;15(3). 10.3390/cancers15030819. 10.3390/cancers15030819 PMC9913844 36765778

[82] Hill M, Pereira V, Chauveau C, Zagani R, Remy S, Tesson L, et al. Heme oxygenase-1 inhibits rat and human breast cancer cell proliferation: mutual cross inhibition with indoleamine 2,3-dioxygenase. Faseb j. 2005;19(14):1957–68. 10.1096/fj.05-3875com. 16319139 10.1096/fj.05-3875com

[83] Gandini NA, Alonso EN, Fermento ME, Mascaró M, Abba MC, Coló GP, et al. Heme Oxygenase-1 Has an Antitumor Role in Breast Cancer. Antioxid Redox Signal. 2019;30(18):2030–49. 10.1089/ars.2018.7554. 30484334 10.1089/ars.2018.7554

[84] Maralbashi S, Aslan C, Kahroba H, Asadi M, Soltani-Zangbar MS, Haghnavaz N, et al. Docosahexaenoic acid (DHA) impairs hypoxia-induced cellular and exosomal overexpression of immune-checkpoints and immunomodulatory molecules in different subtypes of breast cancer cells. BMC Nutr. 2024;10(1):41. 10.1186/s40795-024-00844-y. 38439112 10.1186/s40795-024-00844-y PMC10910708

[85] Chung H, Lee YS, Mayoral R, Oh DY, Siu JT, Webster NJ, et al. Omega-3 fatty acids reduce obesity-induced tumor progression independent of GPR120 in a mouse model of postmenopausal breast cancer. Oncogene. 2015;34(27):3504–13. 10.1038/onc.2014.283. 25220417 10.1038/onc.2014.283 PMC4362785

[86] Dyari HRE, Rawling T, Chen Y, Sudarmana W, Bourget K, Dwyer JM, et al. A novel synthetic analogue of ω-3 17,18-epoxyeicosatetraenoic acid activates TNF receptor-1/ASK1/JNK signaling to promote apoptosis in human breast cancer cells. Faseb j. 2017;31(12):5246–57. 10.1096/fj.201700033R. 28798154 10.1096/fj.201700033R

[87] El-Mesery ME, Al-Gayyar MM, Salem HA, Darweish MM, El-Mowafy AM. Chemopreventive and renal protective effects for docosahexaenoic acid (DHA): implications of CRP and lipid peroxides. Cell Div. 2009;4:17. 10.1186/1747-1028-4-6. 19341447 10.1186/1747-1028-4-6 PMC2680397

[88] Guo CH, Hsia S, Chung CH, Lin YC, Shih MY, Chen PC, et al. Combination of Fish Oil and Selenium Enhances Anticancer Efficacy and Targets Multiple Signaling Pathways in Anti-VEGF Agent Treated-TNBC Tumor-Bearing Mice. Mar Drugs. 2021;19(4). 10.3390/md19040193. 10.3390/md19040193 PMC8065403 33805447

[89] Hamid R, Singh J, Reddy BS, Cohen LA. Inhibition by dietary menhaden oil of cyclooxygenase-1 and – 2 in N-nitrosomethylurea-induced rat mammary tumors. Int J Oncol. 1999;14(3):523–8. 10.3892/ijo.14.3.523. 10024686 10.3892/ijo.14.3.523

[90] Ion G, Akinsete JA, Witte TR, Bostan M, Hardman WE. Maternal fish oil consumption has a negative impact on mammary gland tumorigenesis in C3(1) Tag mice offspring. Eur J Nutr. 2021;60(7):3771–81. 10.1007/s00394-021-02546-8. 33817748 10.1007/s00394-021-02546-8 PMC8440340

[91] Manni A, Xu H, Washington S, Aliaga C, Das A, Cooper T, et al. The effects of Tamoxifen and fish oil on mammary carcinogenesis in polyoma middle T transgenic mice. Horm Cancer. 2011;2(4):249–59. 10.1007/s12672-011-0078-2. 21769696 10.1007/s12672-011-0078-2 PMC10358085

[92] Da Silva NCR, Silva YD, Takahashi JA, Andrade SP, Cassali GD, De Oliveira DR. Dietary lipids management: are there any benefits from the prevention and treatment of 4T1 murine breast carcinoma? Acad Bras Cienc. 2023;95(2):18. 10.1590/0001-3765202320210330. 10.1590/0001-3765202320210330 37585893

[93] Alquobaili F, Miller SA, Muhie S, Day A, Jett M, Hammamieh R. Estrogen receptor-dependent genomic expression profiles in breast cancer cells in response to fatty acids. J Carcinog. 2010;8:17. 10.4103/1477-3163.59539. 20336194 10.4103/1477-3163.59539 PMC2844056

[94] Altenburg JD, Siddiqui RA. Omega-3 polyunsaturated fatty acids down-modulate CXCR4 expression and function in MDA-MB-231 breast cancer cells. Mol Cancer Res. 2009;7(7):1013–20. 10.1158/1541-7786.mcr-08-0385. 19567784 10.1158/1541-7786.MCR-08-0385

[95] Altenburg JD, Bieberich AA, Terry C, Harvey KA, Vanhorn JF, Xu Z, et al. A synergistic antiproliferation effect of curcumin and docosahexaenoic acid in SK-BR-3 breast cancer cells: unique signaling not explained by the effects of either compound alone. BMC Cancer. 2011;11:149. 10.1186/1471-2407-11-149. 21510869 10.1186/1471-2407-11-149 PMC3111403

[96] Ghaffari-Makhmalbaf P, Sayyad M, Pakravan K, Razmara E, Bitaraf A, Bakhshinejad B, et al. Docosahexaenoic acid reverses the promoting effects of breast tumor cell-derived exosomes on endothelial cell migration and angiogenesis. Life Sci. 2021;264:118719. 10.1016/j.lfs.2020.118719. 33159957 10.1016/j.lfs.2020.118719

[97] Hammamieh R, Chakraborty N, Miller SA, Waddy E, Barmada M, Das R, et al. Differential effects of omega-3 and omega-6 Fatty acids on gene expression in breast cancer cells. Breast Cancer Res Treat. 2007;101(1):7–16. 10.1007/s10549-006-9269-x. 16823509 10.1007/s10549-006-9269-x

[98] Hannafon BN, Carpenter KJ, Berry WL, Janknecht R, Dooley WC, Ding WQ. Exosome-mediated microRNA signaling from breast cancer cells is altered by the anti-angiogenesis agent docosahexaenoic acid (DHA). Mol Cancer. 2015;14:133. 10.1186/s12943-015-0400-7. 26178901 10.1186/s12943-015-0400-7 PMC4504101

[99] Hwang JK, Yu HN, Noh EM, Kim JM, Hong OY, Youn HJ, et al. DHA blocks TPA-induced cell invasion by inhibiting MMP-9 expression via suppression of the PPAR-γ/NF-κB pathway in MCF-7 cells. Oncol Lett. 2017;13(1):243–9. 10.3892/ol.2016.5382. 28123548 10.3892/ol.2016.5382 PMC5245160

[100] Newell M, Brun M, Field CJ. Treatment with DHA Modifies the Response of MDA-MB-231 Breast Cancer Cells and Tumors from nu/nu Mice to Doxorubicin through Apoptosis and Cell Cycle Arrest. J Nutr. 2019;149(1):46–56. 10.1093/jn/nxy224. 30601995 10.1093/jn/nxy224

[101] Pizato N, Luzete BC, Kiffer L, Corrêa LH, de Oliveira Santos I, Assumpção JAF, et al. Omega-3 docosahexaenoic acid induces pyroptosis cell death in triple-negative breast cancer cells. Sci Rep. 2018;8(1):1952. 10.1038/s41598-018-20422-0. 29386662 10.1038/s41598-018-20422-0 PMC5792438

[102] Wang L, Choi HS, Lee B, Choi JH, Jang YS, Seo JW. 13R,20-Dihydroxydocosahexaenoic Acid, a Novel Dihydroxy- DHA Derivative, Inhibits Breast Cancer Stemness through Regulation of the Stat3/IL-6 Signaling Pathway by Inducing ROS Production. Antioxid (Basel). 2021;10(3). 10.3390/antiox10030457. 10.3390/antiox10030457 PMC7999786 33804152

[103] Du A, Wang Z, Huang T, Xue S, Jiang C, Qiu G, et al. Fatty acids in cancer: Metabolic functions and potential treatment. MedComm – Oncol. 2023;2(1):e25. 10.1002/mog2.25.

[104] Ding XZ, Hennig R, Adrian TE. Lipoxygenase and cyclooxygenase metabolism: new insights in treatment and chemoprevention of pancreatic cancer. Mol Cancer. 2003;2:10. 10.1186/1476-4598-2-10. 12575899 10.1186/1476-4598-2-10 PMC149414

[105] Calder PC. Omega-3 fatty acids and inflammatory processes: from molecules to man. Biochem Soc Trans. 2017;45(5):1105–15. 10.1042/bst20160474. 28900017 10.1042/BST20160474

[106] de Bus I, Witkamp R, Zuilhof H, Albada B, Balvers M. The role of n-3 PUFA-derived fatty acid derivatives and their oxygenated metabolites in the modulation of inflammation. Prostaglandins Other Lipid mediat. 2019;144:106351. 10.1016/j.prostaglandins.2019.106351. 31260750 10.1016/j.prostaglandins.2019.106351

[107] Jin K, Qian C, Lin J, Liu B. Cyclooxygenase-2-Prostaglandin E2 pathway: A key player in tumor-associated immune cells. Front Oncol. 2023;13:1099811. 10.3389/fonc.2023.1099811. 36776289 10.3389/fonc.2023.1099811 PMC9911818

[108] Calder PC. Omega-3 fatty acids and inflammatory processes. Nutrients. 2010;2(3):355–74. 10.3390/nu2030355. 22254027 10.3390/nu2030355 PMC3257651

[109] Hart PC, Rajab IM, Alebraheem M, Potempa LA. C-Reactive Protein and Cancer-Diagnostic and Therapeutic Insights. Front Immunol. 2020;11:595835. 10.3389/fimmu.2020.595835. 33324413 10.3389/fimmu.2020.595835 PMC7727277

[110] Platz EA, De Marzo AM, Erlinger TP, Rifai N, Visvanathan K, Hoffman SC, et al. No association between pre-diagnostic plasma C-reactive protein concentration and subsequent prostate cancer. Prostate. 2004;59(4):393–400. 10.1002/pros.10368. 15065087 10.1002/pros.10368

[111] Cabral-Pacheco GA, Garza-Veloz I, Castruita-De la Rosa C, Ramirez-Acuña JM, Perez-Romero BA, Guerrero-Rodriguez JF, et al. The Roles of Matrix Metalloproteinases and Their Inhibitors in Human Diseases. Int J Mol Sci. 2020;21(24). 10.3390/ijms21249739. 10.3390/ijms21249739 PMC7767220 33419373

[112] Li H, Qiu Z, Li F, Wang C. The relationship between MMP-2 and MMP-9 expression levels with breast cancer incidence and prognosis. Oncol Lett. 2017;14(5):5865–70. 10.3892/ol.2017.6924. 29113219 10.3892/ol.2017.6924 PMC5661385

[113] Jiang H, Li H. Prognostic values of tumoral MMP2 and MMP9 overexpression in breast cancer: a systematic review and meta-analysis. BMC Cancer. 2021;21(1):149. 10.1186/s12885-021-07860-2. 33568081 10.1186/s12885-021-07860-2 PMC7877076

[114] Poland M, Ten Klooster JP, Wang Z, Pieters R, Boekschoten M, Witkamp R, et al. Docosahexaenoyl serotonin, an endogenously formed n-3 fatty acid-serotonin conjugate has anti-inflammatory properties by attenuating IL-23-IL-17 signaling in macrophages. Biochim Biophys Acta. 2016;1861(12 Pt A):2020–8. 10.1016/j.bbalip.2016.09.012. 27663185 10.1016/j.bbalip.2016.09.012

[115] de Visser KE, Joyce JA. The evolving tumor microenvironment: From cancer initiation to metastatic outgrowth. Cancer Cell. 2023;41(3):374–403. 10.1016/j.ccell.2023.02.016. 36917948 10.1016/j.ccell.2023.02.016

[116] Kwon Y, Kim M, Kim Y, Jung HS, Jeoung D. Exosomal MicroRNAs as Mediators of Cellular Interactions Between Cancer Cells and Macrophages. Front Immunol. 2020;11:1167. 10.3389/fimmu.2020.01167. 32595638 10.3389/fimmu.2020.01167 PMC7300210

[117] Stefanovska B, André F, Fromigué O. Tribbles Pseudokinase 3 Regulation and Contribution to Cancer. Cancers (Basel). 2021;13(8). 10.3390/cancers13081822. 10.3390/cancers13081822 PMC8070086 33920424

[118] Arif A, Alameri AA, Tariq UB, Ansari SA, Sakr HI, Qasim MT, et al. The functions and molecular mechanisms of Tribbles homolog 3 (TRIB3) implicated in the pathophysiology of cancer. Int Immunopharmacol. 2023;114:109581. 10.1016/j.intimp.2022.109581. 36527874 10.1016/j.intimp.2022.109581

[119] Laverty HG, Wakefield LM, Occleston NL, O’Kane S, Ferguson MW. TGF-beta3 and cancer: a review. Cytokine Growth Factor Rev. 2009;20(4):305–17. 10.1016/j.cytogfr.2009.07.002. 19656717 10.1016/j.cytogfr.2009.07.002 PMC7294566

[120] Liu D, Zhang P, Zhou J, Liao R, Che Y, Gao M-M, et al. TNFAIP3 Interacting Protein 3 Overexpression Suppresses Nonalcoholic Steatohepatitis by Blocking TAK1 Activation. Cell Metabol. 2020;31(4):726–e408. 10.1016/j.cmet.2020.03.007. 10.1016/j.cmet.2020.03.007 32268115

[121] Nitti M, Ivaldo C, Traverso N, Furfaro AL. Clinical Significance of Heme Oxygenase 1 in Tumor Progression. Antioxid (Basel). 2021;10(5). 10.3390/antiox10050789. 10.3390/antiox10050789 PMC8155918 34067625

[122] Green JE, Shibata M-A, Yoshidome K, Liu M-l, Jorcyk C, Anver MR, et al. The C3(1)/SV40 T-antigen transgenic mouse model of mammary cancer: ductal epithelial cell targeting with multistage progression to carcinoma. Oncogene. 2000;19(8):1020–7. 10.1038/sj.onc.1203280. 10713685 10.1038/sj.onc.1203280

[123] Lee HS. Impact of Maternal Diet on the Epigenome during In Utero Life and the Developmental Programming of Diseases in Childhood and Adulthood. Nutrients. 2015;7(11):9492–507. 10.3390/nu7115467. 26593940 10.3390/nu7115467 PMC4663595

[124] Godfrey KM, Gluckman PD, Hanson MA. Developmental origins of metabolic disease: life course and intergenerational perspectives. Trends Endocrinol Metabolism. 2010;21(4):199–205. 10.1016/j.tem.2009.12.008. 10.1016/j.tem.2009.12.008 20080045

[125] Abbas A, Witte T, Patterson WL, Fahrmann JF, Guo K, Hur J, et al. Epigenetic Reprogramming Mediated by Maternal Diet Rich in Omega-3 Fatty Acids Protects From Breast Cancer Development in F1 Offspring. Front Cell Dev Biol. 2021;9:10. 10.3389/fcell.2021.682593. 10.3389/fcell.2021.682593 PMC8222782 34179012

[126] Xing S, Gai K, Li X, Shao P, Zeng Z, Zhao X, et al. Tcf1 and Lef1 are required for the immunosuppressive function of regulatory T cells. J Exp Med. 2019;216(4):847–66. 10.1084/jem.20182010. 30837262 10.1084/jem.20182010 PMC6446865

[127] Santiago L, Daniels G, Wang D, Deng FM, Lee P. Wnt signaling pathway protein LEF1 in cancer, as a biomarker for prognosis and a target for treatment. Am J Cancer Res. 2017;7(6):1389–406. 28670499 PMC5489786

[128] Huang FI, Chen YL, Chang CN, Yuan RH, Jeng YM. Hepatocyte growth factor activates Wnt pathway by transcriptional activation of LEF1 to facilitate tumor invasion. Carcinogenesis. 2012;33(6):1142–8. 10.1093/carcin/bgs131. 22436613 10.1093/carcin/bgs131

[129] Lima BM, Azevedo ALKd, Giner IS, Gomig THB, Ribeiro EMSF, Cavalli IJ. Biomarker potential of the LEF1/TCF family members in breast cancer: Bioinformatic investigation on expression and clinical significance. Genet Mol Biology. 2023;46(4):e20220346. 10.1590/1678-4685-GMB-2022-0346 PMC10723634 38100720

[130] Rodriguez-Ubreva J, Ciudad L, van Oevelen C, Parra M, Graf T, Ballestar E. C/EBPa-mediated activation of microRNAs 34a and 223 inhibits Lef1 expression to achieve efficient reprogramming into macrophages. Mol Cell Biol. 2014;34(6):1145–57. 10.1128/mcb.01487-13. 24421386 10.1128/MCB.01487-13 PMC3958044

[131] Heino S, Fang S, Lähde M, Högström J, Nassiri S, Campbell A, et al. Lef1 restricts ectopic crypt formation and tumor cell growth in intestinal adenomas. Sci Adv. 2021;7(47):eabj0512. 10.1126/sciadv.abj0512. 34788095 10.1126/sciadv.abj0512 PMC8598008

[132] Song P, Gao Z, Bao Y, Chen L, Huang Y, Liu Y, et al. Wnt/β-catenin signaling pathway in carcinogenesis and cancer therapy. J Hematol Oncol. 2024;17(1):46. 10.1186/s13045-024-01563-4. 38886806 10.1186/s13045-024-01563-4 PMC11184729

[133] Ishihara T, Yoshida M, Arita M. Omega-3 fatty acid-derived mediators that control inflammation and tissue homeostasis. Int Immunol. 2019;31(9):559–67. 10.1093/intimm/dxz001. 30772915 10.1093/intimm/dxz001

[134] Westheim AJF, Stoffels LM, Dubois LJ, van Bergenhenegouwen J, van Helvoort A, Langen RCJ, et al. Fatty Acids as a Tool to Boost Cancer Immunotherapy Efficacy. Front Nutr. 2022;9:868436. 10.3389/fnut.2022.868436. 35811951 10.3389/fnut.2022.868436 PMC9260274

[135] Marchio V, Augimeri G, Morelli C, Vivacqua A, Giordano C, Catalano S, et al. Omega-3 fatty acids: molecular weapons against chemoresistance in breast cancer. Cell Mol Biol Lett. 2025;30(1):11. 10.1186/s11658-025-00694-x. 39863855 10.1186/s11658-025-00694-x PMC11762563

[136] Murphy RA, Mourtzakis M, Chu QS, Baracos VE, Reiman T, Mazurak VC. Supplementation with fish oil increases first-line chemotherapy efficacy in patients with advanced nonsmall cell lung cancer. Cancer. 2011;117(16):3774–80. 10.1002/cncr.25933. 21328326 10.1002/cncr.25933

[137] Ibrahim A, Mohamady Farouk Abdalsalam N, Liang Z, Kashaf Tariq H, Li R, Afolabi O. MDSC checkpoint blockade therapy: a new breakthrough point overcoming immunosuppression in cancer immunotherapy. Cancer Gene Ther. 2025;32(4):371–92. 10.1038/s41417-025-00886-9. 40140724 10.1038/s41417-025-00886-9 PMC11976280

