# Supplementary material for: The Role of Omega-3 Polyunsaturated Fatty Acids in Modulating Inflammation and Breast Cancer: A Systematic Review of In Vitro, Animal, and Human Studies
Source: Curr Oncol Rep. 2026 Aug 21;28(1):83. doi: 10.1007/s11912-026-01820-4 (PMC13498503; doi:10.1007/s11912-026-01820-4)
Supplement: Supplementary file 1 [file 11912_2026_1820_MOESM1_ESM.docx]

**The Role of Omega-3 Polyunsaturated Fatty Acids in Modulating Inflammation and Breast Cancer: A Systematic Review of In Vitro, Animal, and Human Studies**

Rahbika Ashraf^1^, Connor D.C. Buchanan^1^, Jennifer M. Monk^1^, Lindsay E. Robinson^1^, David W.L. Ma^1^*

^1^Department of Human Health Sciences, University of Guelph, Guelph, Ontario, N1G 2W1, Canada

***Correspondence:**

David W. L. Ma, davidma@uoguelph.ca

50 Stone Road E

Guelph, Ontario, Canada

N1G 2W1

**Supplementary Material**

**Table S1: PubMed Search Strategy**

| **Search Component** | **Search Terms** | **Filters Applied** | **Articles Retrieved** |
| --- | --- | --- | --- |
| **n-3 PUFA** | "Fatty Acids, Omega-3"[Mesh] OR "Fish Oils"[Mesh] OR "Dietary Fats"[Mesh] OR "Eicosapentaenoic Acid"[Mesh] OR "Docosahexaenoic Acids"[Mesh] OR "alpha-Linolenic Acid"[Mesh] OR "Omega-3"[Title/Abstract] OR "n-3 PUFA"[Title/Abstract] OR "n-3 Fatty Acids"[Title/Abstract] OR "EPA"[Title/Abstract] OR "DHA"[Title/Abstract] OR "ALA"[Title/Abstract] | Full text, English, Exclude preprints | 152,899 |
| **Breast Cancer** | "Breast Neoplasms"[Mesh] OR "Breast Cancer"[Title/Abstract] OR "Breast Tumour"[Title/Abstract] OR "Breast Tumor"[Title/Abstract] OR "Mammary Cancer"[Title/Abstract] OR "Cancer of the Breast"[Title/Abstract] | Full text, English, Exclude preprints | 409,224 |
| **Inflammation** | "Inflammation" OR "Inflammation Mediators" OR "Immune Cells" OR "Immune System" OR "Leukocytes" OR "Lymphocytes" OR "Macrophages" OR "Neutrophils" OR "Monocytes" OR "Antigen-Presenting Cells" OR "T Cells" OR "B Cells" OR "Cytokines" OR "Chemokines" OR "Interleukins" OR "Prostaglandins" OR "Leukotrienes" OR "Tumor Necrosis Factors" OR “Reactive Oxygen Species" OR "Nitric Oxide" | Full text, English, Exclude preprints | 3,432,647 |
| **Excluded Articles** | NOT ((Systematic Review[Publication Type]) OR (Meta-Analysis[Publication Type]) OR (Review[Publication Type])) | Full text, English, Exclude preprints | 3,029,500 |
| **Combined Search** | ((n-3 PUFA terms) AND (Breast Cancer terms) AND (Inflammation terms)) | Full text, English, Exclude preprints | 1,713 |
| **Final Articles for Review** | Combined search with all filters applied:  (((("Fatty Acids, Omega-3"[Mesh] OR "Fish Oils"[Mesh] OR "Dietary Fats"[Mesh] OR "Eicosapentaenoic Acid"[Mesh] OR "Docosahexaenoic Acids"[Mesh] OR "alpha-Linolenic Acid"[Mesh] OR "Omega-3"[Title/Abstract] OR "n-3 PUFA"[Title/Abstract] OR "n-3 Fatty Acids"[Title/Abstract] OR "Fish Oils"[Title/Abstract] OR "Eicosapentaenoic Acid"[Title/Abstract] OR "EPA"[Title/Abstract] OR "Docosahexaenoic Acid"[Title/Abstract] OR "DHA"[Title/Abstract] OR "Alpha-Linolenic"[Title/Abstract] OR "ALA"[Title/Abstract]) AND ((excludepreprints[Filter]) AND (fft[Filter]) AND (english[Filter]))) AND (("Breast Neoplasms"[Mesh] OR "Breast Cancer"[Title/Abstract] OR "Breast Tumour"[Title/Abstract] OR "Breast Tumor"[Title/Abstract] OR "Mammary Cancer" [Title/Abstract] OR "Cancer of the Breast"[Title/Abstract]) AND ((excludepreprints[Filter]) AND (fft[Filter]) AND (english[Filter])))) AND ((((((((((((((((((((Inflammation) OR (Inflammation Mediators)) OR (Immune Cells)) OR (Immune System)) OR (Leukocytes)) OR (Lymphocytes)) OR (Macrophages)) OR (Neutrophils)) OR (Monocytes)) OR (Antigen-Presenting Cells)) OR (T Cells)) OR (B Cells)) OR (Cytokines)) OR (Chemokines)) OR (Interleukins)) OR (Prostaglandins)) OR (Leukotrienes)) OR (Tumor Necrosis Factors)) OR (Reactive Oxygen Species)) OR (Nitric Oxide) AND ((excludepreprints[Filter]) AND (fft[Filter]) AND (english[Filter]))) AND ((excludepreprints[Filter]) AND (fft[Filter]) AND (english[Filter]))) NOT (((Systematic Review[Publication Type]) OR (Meta-Analysis[Publication Type])) OR (Review[Publication Type]) AND ((excludepreprints[Filter]) AND (fft[Filter]) AND (english[Filter]))) Filters: Full text, English, Exclude preprints, from 1977 – 2025 | Full text, English, Exclude preprints, 1977–2025 | 366 |

[Title/Abstract]- restrict the query to search in the title or abstract of the articles; [mesh]- Medical Subject Headings.

**Table S2:** Web of Science Search Strategy

| **Search Component** | **Search Terms** | **Filters Applied** | **Articles Retrieved** |
| --- | --- | --- | --- |
| **n-3 PUFA** | TS=(Omega-3 fatty acids) OR TS=(n-3 PUFA) OR TS=(fish oils) OR TS=(EPA) OR TS=(DHA) OR TS=(Eicosapentaenoic Acid) OR TS=(Docosahexaenoic Acids) OR TS=(Alpha-Linolenic Acid) OR TS=(ALA) | Languages: English | 205,955 |
| **Breast Cancer** | TS=(breast cancer) OR TS=(breast neoplasms) OR TS=(breast tumour) OR TS=(breast tumor) OR TS=(mammary cancer) | Languages: English | 796,339 |
| **Inflammation / Immune System** | TS=(inflammation) OR TS=(immune system) | Languages: English | 1,226,288 |
| **Combined Search** | (n-3 PUFA terms) AND (Breast Cancer terms) AND (Inflammation / Immune System terms) | Languages: English | 203 |
| **Refine** | NOT Document Types: Review Article, Book Chapter, Meeting Abstract, Editorial Material; Document Types: Article | Languages: English; Document Types: Article | 195 |
| **Final Articles** | Combined search after applying all filters | Refined by Document Type and Language | 195 |
